# Tumor cell endogenous HIF-1α activity induces aberrant angiogenesis and interacts with TRAF6 pathway required for colorectal cancer development

**DOI:** 10.1016/j.neo.2020.10.006

**Published:** 2020-10-24

**Authors:** Jesus F. Glaus Garzon, Chiara Pastrello, Igor Jurisica, Michael O. Hottiger, Roland H. Wenger, Lubor Borsig

**Affiliations:** a Institute of Physiology, University of Zurich, Zurich, Switzerland; b Krembil Research Institute, UHN, Toronto, ON, Canada; c Departments of Medical Biophysics and Computer Science, University of Toronto, Toronto, ON, Canada; d Department of Molecular Mechanism of Disease, University of Zurich, Zurich, Switzerland; e Comprehensive Cancer Center Zurich, Zurich, Switzerland

**Keywords:** Colorectal cancer, Tumorigenesis, Tumor hypoxia, HIF-1α, Inflammation, TRAF6

## Abstract

Hypoxia and inflammation are key factors for colorectal cancer tumorigenesis. The colonic epithelium belongs to the tissues with the lowest partial pressure of oxygen in the body, and chronic inflammation is associated with an increased chance to develop colon cancer. How the colonic epithelium responds to hypoxia and inflammation during tumorigenesis remains to be elucidated. Here we show, that murine colon adenocarcinoma cells with attenuated response to hypoxia, due to a knock-down (KD) of HIF-1α, produce smaller and less hypoxic tumors in an orthotopic mouse model when compared to tumors induced with control cells. HIF-1α-KD tumors showed more functional perfused vasculature associated with increased levels of vessel-stabilizing factors and reduced levels of proangiogenic factors, including extracellular matrix protein Cyr61/CCN1. Intratumoral injection of Cyr61 in HIF-1α-KD tumors revealed an in increased vessel permeability and tumor hypoxia. Further bioinformatics analysis identified a possible interaction between HIF-1α and TRAF6, an upstream effector of the NF-κB pathway that was confirmed by coimmunoprecipitation in MC-38 and CT26 colon adenocarcinoma cells and in situ by proximity ligation assay. Down-regulation of TRAF6 resulted in virtual abrogation of orthotopic tumor growth. Subcutaneous TRAF6-KD tumors were smaller and contained reduced vessel size and differently polarized macrophages. These data demonstrate that the tumor cell response to increased hypoxia in the colon leads to promotion of nonfunctional angiogenesis, regulated by both hypoxia and TRAF6 pathways.

## Highlights

• Findings provide evidence that hypoxia response deficient tumors show more functionally perfused vasculature and that TRAF6, an upstream effector of NF-κB, is directly interacting with HIF-1α thereby contributing to enhanced angiogenesis.

## Introduction

Colorectal cancer is the second and third most frequently occurring cancer in women and men, respectively [1]. Hypoxia and inflammation promote tumorigenesis and metastasis through modulation of the tumor microenvironment. Tumor-promoting inflammation contributes to stromal cell polarization supporting angiogenesis [2]. During tumorigenesis, vessels are frequently structurally and functionally abnormal and leaky, which results in reduced perfusion that contributes to enlargement of hypoxic regions, which promote metastasis but also render tumors resistant to chemo- and radiotherapy [3].

Hypoxia-inducible factor (HIF) is the main regulator of the hypoxic response of tumor cells. HIF is a heterodimer constituted by either HIF-1α or HIF-2α and a common HIF-1β subunit. Under normoxia, the HIF-α subunits are modified by prolyl hydroxylases and destined for proteosomal degradation. Under hypoxia, HIF-α subunits are rapidly stabilized and translocate to the nucleus, where upon heterodimerization bind to hypoxia response elements, leading to enhance transcription of several hundred target genes [4,5]. Hypoxia and other factors associated with pathological stress, such as inflammation or cancer, trigger HIF expression, stabilization, and activity in immune cells [6]. Tumor cells also directly react to hypoxia through HIF-driven responses and modulate tumor microenvironment, angiogenesis, and inflammation; thereby promoting tumorigenesis. For instance, HIF-1α expression in glioblastoma tumors is responsible for upregulation of chemokines, e.g., CXCL12 that drives bone marrow-derived myeloid cell recruitment, contributing to vascular remodeling [7]. In breast cancer cells, HIF-1α stabilization induces the expression of the extracellular matrix remodeling enzymes lysyl oxidase and MMPs, leading to a more aggressive phenotype and metastasis [8]. However, in colon cancer HIF-1α and HIF-2α apparently have different roles, regulating cell proliferation and anchor-independent growth, respectively [9]. While, the epithelial disruption of HIF-2α significantly decreased neutrophil infiltration in colon cancer [10], HIF-1α stabilization in proximal colon augments inflammation and cancer progression [11].

Chronic inflammatory diseases, including inflammatory bowel disease and Crohn's disease, increase the probability of developing colorectal cancer [12]. Infiltration of immune cells, such as granulocytes, results in increased production of reactive oxygen species that induces genomic instability and results in malignant transformation and inflammatory activation of epithelial cells [13]. The nuclear factor NF-κB is the central transcription factor activated by a variety of inflammatory factors derived from either tumor cells or a tumor microenvironment during malignant progression [14]. The expression of tumor promoting cytokines, such as IL-6 or TNF-α, and antiapoptosis survival genes are dependent on NF-κB, thus making it a critical factor in cancer progression [14,15]. Altered NF-κB activation has been detected in many solid tumors where it regulates the expression of genes affecting proliferation, migration, and apoptosis [14,16]. Tumor initiation in colorectal cancer is dependent on an intact IKKβ-mediated NF-κB signaling [17]. However, the impact of upstream NF-κB mediators such as IKK in colon cancer remains to be defined.

Microbial oxygen consumption and large oxygen diffusion distances lead to an anoxic environment in the lumen of the colon [18]. Therefore, hypoxia is likely a main modulator of cancer progression in the colon epithelium, which together with the omnipresent inflammatory stimuli derived from microbiota promotes tumor inflammation. Previously, we have shown that MC-38 cells express only the HIF-1α subunit, which is the sole driver of hypoxic responses in this cell line [19]. Here we tested the role of HIF-1α in tumor cells on colorectal tumor development in an orthotopic mouse model using MC-38 mouse adenocarcinoma cells, where the stromal compartment has normal HIF-1α activity. Bioinformatics approaches identified a direct interaction between hypoxic and inflammatory signaling pathways required for tumor progression.

## Materials and methods

### Cell culture

Mouse colon adenocarcinoma cells expressing GFP (MC-38GFP) and CT26 cells were cultured in high glucose DMEM (Sigma) supplemented with 10%FBS, NEAA and 1 mmol/L Na-pyruvate (all Gibco). Cells exposed to hypoxia were grown in a gas-controlled workstation (InvivO_2_ 400, Baker-Ruskinn Technologies). MC-38-HIF-1α-KD cells were prepared as previously described [19]. MC-38-TRAF6-KD was prepared by lentiviral transduction of shRNA vectors in pLKO.1-puro plasmid (Sigma). shRNA constructs targeting mouse TRAF6 and nontarget controls (Mock) were purchased from Sigma.

### Mice

All animal experiments were performed according to the guidelines of the Swiss Animal Protection Law and approved by the Veterinary Office of the Kanton Zurich. C57BL/6 J mice were purchased from Charles River, Germany.

### Mouse tumor models

Orthotopic colon tumor model: mice were anesthetized by intraperitoneal injection of ketamine/xylaxine/acepromazine (65/10/2 mg/kg per mouse) in saline buffer. After shaving of the abdominal part, a small incision (2–3 mm) was performed and the cecum was exposed. Using a 27 G needle, 40,000 tumor cells in growth factor-reduced Matrigel (Corning) were injected in the cecum wall and both peritoneal and skin layers were subsequently stitched. For analgesia, mice were subcutaneously injected with Meloxicam, 5 mg/kg (Boehringer Ingelheim) 30 min prior- and 6 h and 24 h postsurgery. Subcutaneous tumor model: mice were injected in the right flank with 500,000 MC-38GFP Mock or TRAF6-KD cells. Tumor size was measured with caliper every day and mice were sacrificed at day 19 after injection or when the tumor volume reached 1 cm^3^.

### Flow cytometry analysis of tumors

Mice were perfused with phosphate buffer saline (PBS) through the left ventricle and dissected orthotopic tumors were digested with collagenase IV, hyaluronidase and DNAse I (all Sigma) in 2% fetal bovine serum/RPMI (FBS/RPMI) for 1 h at 37 °C. The cell suspension was filtered through a 100-µm cell strainer, erythrocytes lysed, and filtered through a 40-µm cell strainer (BD). Cells were stained with Zombie Fixable Viability Kit (Biolegend) and incubated with anti-CD16/32 for 10 min in FACS buffer (PBS/10 mmol/L EDTA, 2% FCS), followed by incubation with antibodies: anti-CD45 (clone 30-F11), anti-CD11b (clone M1/70), anti-Ly6G (clone 1A8), anti-Ly6C (clone HK1.4); all from Biolegend. In case of subcutaneous tumors the following antibodies were used in addition: anti-CD11c (clone N418), anti-CD64 (clone X54–5/7.1), anti-CD103 (clone 2E7), anti-MHCII (clone M5/114.15.2), anti-CD206 (clone C068C2) and anti-VCAM1 (clone 429); all from Biolegend. Data were acquired with a LSR II Fortessa cytometer (BD) and analyzed by FlowJo software v. 7.6.5 (TreeStar).

### Hypoxia staining and angiogenic analysis of tumors

Mice were i.v. injected with 1.5 mg pimonidazole (Hypoxyprobe) one hour before termination. For determination of tissue perfusion, mice were i.v. injected with 100 µg FITC-labeled *Lycopersicon esculentum* (tomato) lectin (Vector Labs) 5 min prior to whole body perfusion with PBS followed by tumor dissection and embedding in OCT (TissueTek). Tissue sections (5 µm) were fixed for 10 min in ice-cold acetone, rehydrated in PBS and blocked with 1%BSA in PBST (PBS/0.1% Tween 20) for one hour at room temperature (RT). Sections were then incubated with anti-pimonidazole (Omni Kit Hypoxyprobe) and anti-CD31 (Biolegend) antibodies overnight at 4 °C in blocking buffer. After washing with PBST (3×), antibodies: anti-rabbit-AF568, anti-rat-AF647 (Life Technologies) were incubated for 1 h at RT, counterstained with DAPI (Sigma) and mounted in Prolong Gold (Life Technologies). Pericytes were stained with anti-NG2 (Millipore). Pimonidazole, CD31 or NG2 staining was determined using MIRAX MIDI Slide Scanner (Zeiss). Images were acquired with a CLSM SP5 Resonant APD Confocal Microscope (Leica) and analyzed with the Imaris software (Bitplane). Vessel area was calculated using the software Pannoramic Viewer 1.15.2 (3D Histech).

### Vascular permeability assay

Mice with orthotopic tumors were intratumorally injected with recombinant Cyr61 (400 ng, Cusabio Biotech) reconstituted in HBSS every other day starting on day 21 after tumor cell injection (3× in total). Prior to termination, mice were injected with pimonidazole (as described above) followed by i.v. injection of Evans Blue (2 mg) and terminated 30 min later; dissected tumors were embedded in OCT. Staining of pimonidazole and CD31 was performed as described above. Evans Blue staining was analyzed using optimal excitation and emission filters at 620 and 680 nm, respectively.

### Cytokine analysis

Cytokines and chemokines in tumor lysates were analyzed using ProcartaPlex Mouse Panel 1 (26-plex; eBioscience) using a Bio-Plex System (Biorad) with xMAP Luminex technology. Protein concentration was determined using the BCA assay following the manufacturer's instructions (Thermo Fisher).

### Gene expression in sorted cells

Cell suspension (described above) was stained with Zombie Fixable Viability Kit followed by incubation with anti-CD16/32 and staining with anti-CD45 (clone 30-F11) and anti-CD31 (clone 390); all from Biolegend. At least 50,000 tumor cells (CD45^neg^,CD31^neg^,GFP^+^) were sorted per sample. RNA was extracted using RNeasy Plus Mini Kit (Qiagen) and cDNA was synthesized using Omniscript RT Kit (Qiagen). qPCR was performed with KAPA SYBR FAST quantitative PCR (qPCR) Master Mix (KAPA Biosystems) and analyzed in a CFX96 Touch Real-Time PCR Detection System (Biorad).

### RNA library preparation

The quantity and the quality of the isolated RNA was determined with a Qubit (1.0) Fluorometer (Life Technologies) and a Bioanalyzer 2100 (Agilent). The TruSeq Stranded mRNA Sample Prep Kit (Illumina) was used in the next steps. Briefly, total RNA samples (100 ng) were poly-A selected and then reverse-transcribed into double-stranded cDNA with Actinomycin D added during first-strand synthesis. The cDNA samples were fragmented, end-repaired and adenylated before ligation of TruSeq adapters. The adapters contain the index for multiplexing. Fragments containing TruSeq adapters on both ends were selectively enriched with PCR. The quality and quantity of the enriched libraries were validated using Qubit (1.0) Fluorometer and the Bioanalyzer 2100.

### Cluster generation and sequencing

The TruSeq SR Cluster Kit v4-cBot-HS (Illumina) was used for cluster generation using 8 pM of pooled normalized libraries on the cBOT. Sequencing were performed on the Illumina HiSeq 4000 single end 125 bp using the TruSeq SBS Kit v4-HS (Illumina).

### RNA sequencing data analysis

Bioinformatics analysis of RNA sequencing data was performed with SUSHI software [20]. In detail, the raw reads were quality checked using Fastqc (http://www.bioinformatics.babraham.ac.uk/projects/fastqc/) and FastQ Screen (http://www.bioinformatics.babraham.ac.uk/projects/fastq_screen/). Quality controlled reads (adaptor trimmed, first 5 and last 6 bases hard trimmed, minimum average quality Q10, minimum tail quality Q10, minimum read length 20 nt) were aligned to the reference genome (Ensembl GRCh38, not patched) using STAR aligner [21]. Read alignments were only reported for reads with less than 50 valid alignments. Expression counts were computed using feature Counts in the Bioconductor package Subread [22]. Differential expression was computed using the DESeq2 package [23]. The RNAseq data have been deposited in gene expression omnibus database under the accession number (GSE155104).

### Protein-interaction network analysis

Differently expressed genes from MC-38 cells cultivated under hypoxia and normoxia were used for further analyses: mouse I2D ver. 2.3 (http://ophid.utoronto.ca/i2d) was used to identify their interacting partners [24]. Resulting network was annotated, analyzed and visualized using NAViGaTOR 3.013 [25]. Gene Ontology enrichment analysis was performed using clusterProfiler_3.16.0 [26] on mouse genome wide annotation (org.Mm.eg.db 3.11.4). Enrichment was performed on Biological Process – BP and Cellular Component – CC. Top 20 terms from each of the ontology were plotted. Mouse gene symbols were converted to human gene symbols using biomaRt 2.44.0 [27], and 568 mouse gene symbols were converted to 526 human gene symbols. Disease enrichment analysis was performed on the list of human gene symbols with DOSE_3.14.0 [28] using DisGeNet (DGN). Plots were obtained using clusterProfiler. All analyses were performed using R 4.0.0. (https://www.r-project.org/).

### Immunoprecipitation

MC-38 Mock, HIF-1α-KD and TRAF6-KD cells were cultured under hypoxia (0.2% O_2_) for 8 hours with or without LPS stimulation (1 µg/mL) for 1 h at 37 °C. Cells were resuspended in lysis buffer (10 mM Tris-HCl pH 8.0, 1 mM EDTA, 400 mM NaCl, 0.1% NP-40) and Sigma's Protease Inhibitor Cocktail. Total protein amount was determined using the BCA assay. Total lysate (1.5 mg) was incubated with 6 µg anti-TRAF6 (Clone: d-10, Santa Cruz) or anti-HIF-1α antibody (Clone: H1α67, Santa Cruz) and 40 µL Dynabead Protein G (ThermoFisher) slurry overnight at 4 °C. Beads were washed with PBS (3×), boiled in Laemmli buffer at 95 °C for 5 min. The supernatant was separated by 10% SDS-PAGE, transferred to a nitrocellulose membrane, and incubated with anti-HIF-1α (rabbit polyclonal, Novus Biologicals) or anti-TRAF6 (Clone: EP591Y, Abcam) antibodies, respectively.

### Immunohistochemistry

Tissue paraffin sections (5 µm) were stained with hematoxylin/eosin or the following antibodies: Ki67 (NeoMarkers), and cleaved caspase-3 (Cell Signaling). Staining was performed on a NEXES immune-histochemistry robot (Ventana Instruments) using an IVIEW DAB Detection Kit (Ventana Instruments) or on a Bond MAX (Leica). Images were digitized with a Axio Scan.Z1 slide scanner (Zeiss) and analyzed using Zen Blue image analysis software (Zeiss) and Fiji (ImageJ). Tissue sections were stained simultaneously for each antigen and the signal-to-noise cutoff was manually adjusted for each antibody staining and applied to all samples within one staining group.

### Proximity ligation assay

MC-38 cells were cultured on coverslips (12 mm diameter) under normoxia (21% O_2_) or hypoxia (0.2% O_2_) for 8 h. Cells were washed with 1× PBS and fixed with 4% paraformaldehyde (PFA) for 15 min at room temperature. Samples were washed twice with 1× PBS and permeabilized with 0.1% Tween-20 in 1× PBS for 10 min at room temperature. Duolink In Situ Orange Starter Kit Mouse/Rabbit (Sigma) was used together with a mouse-anti HIF1α (Clone: H1alpha67, Novus Biologicals) and rabbit-anti TRAF6 (ab33915, Abcam) antibodies were used at 20 µg/mL. Blocking and staining was performed following manufacturer's recommendations.

### Statistical analysis

Statistical analysis was performed with the GraphPad Prism software (version 6.03). All data are presented as mean ±SEM and were analyzed by ANOVA with the post-hoc Bonferroni multiple comparison test. Analysis of 2 groups was performed with Mann-Whitney test unless stated otherwise.

## Results

### Impaired hypoxic response in colorectal tumor cells results in smaller orthotopic tumors

**Figure 1:**
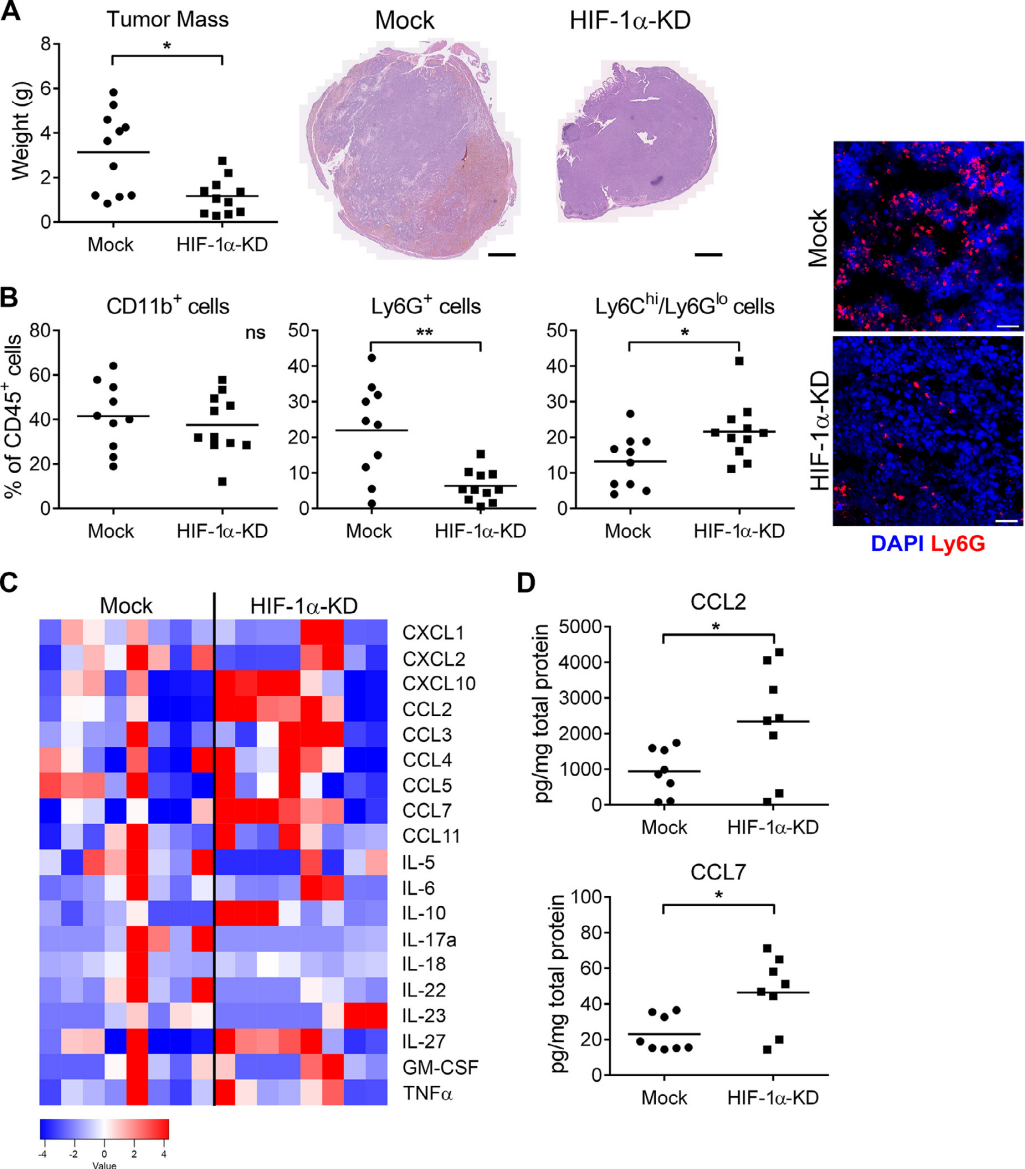
MC-38 colorectal tumor cells with reduced HIF-1α transcript levels lead to reduced orthotopic tumor growth associated with changes in tumor microenvironment. (A) Weight of tumors 28 days after intracecal injection of HIF-1α-KD and control (Mock) MC-38 cells (left panel). Representative images of H&E stained tumors from respective tumors (right panel). Scale bar = 1 mm. (B) Flow cytometry analysis of immune cells in tumors: CD11b^+^/Ly6C^−^/Ly6G^lo^ myeloid cells (left panel); Ly6G^+^ granulocytes (middle panel), Ly6C^hi^/Ly6G^lo^ inflammatory monocytes (right panel) presented as percentage of total living CD45^+^ cells. Representative images of orthotopic tumors stained with Ly6G-antibody (red) and counterstained with DAPI. Bar = 30 µm. (C) Amounts of cytokines detected in tumor homogenates at day 28. (D) Amounts of Ccl2 and Ccl7 chemokines in tumor homogenates. Statistical significance was assessed using the Mann-Whitney test; *, *P* < 0.05; **, *P* < 0.01. (Color version of figure is available online.)

To test how impaired responsiveness of tumor cells to hypoxia affects tumorigenesis, we tested the growth of mouse colon carcinoma cells (MC-38) with stable down-regulation of HIF-1α (HIF-1α-KD) in an orthotopic model. We previously showed that the hypoxic response in MC-38 cells is solely dependent on HIF-1α [19]. We confirmed that MC-38 HIF-1α-KD cells have reduced HIF-1α mRNA levels and minimally respond to hypoxia (Supplementary Figure 1). Intracecal injection of HIF-1α-KD cell resulted in reduced tumor growth when compared to control MC-38 (Mock) cells (Figure 1A). Since hypoxia had previously been shown to attenuate inflammatory responses in MC-38 cells in vitro [19], we analyzed the composition of the immune cells in the tumor microenvironment by flow cytometry. Analysis of tumor infiltrating cells revealed a reduced number of granulocytes (Ly6G^+^ cells), but increased presence of inflammatory monocytes (Ly6C^+^ cells) in HIF-1α-KD tumors, when compared to Mock tumors (Figure 1B). We observed no major changes in the number of macrophages; CD64^+^CD11c^−^; or tumor-associated macrophages; TAMs - CD64^+^CD11c^+^; (Supplementary Figure 1D-E). Neither there were any significant changes in polarization of macrophages or TAMs. Histological analysis of tumors showed a reduced presence of Ly6G^+^ cells. However, no changes in numbers of myeloid cells (CD11b^+^Ly6C^−^Ly6G^lo^) were observed (Figure 1B). To understand the changes in tumor infiltration of innate immune cells caused by reduced HIF-1α activity, we analyzed the levels of cytokines in tumor homogenates (Figure 1C). Increased levels of CCL2 and CCL7 were detected in HIF-1α-KD tumors, which correlated with the enhanced presence of inflammatory monocytes (Figure 1D).

### HIF-1α-KD tumors are less hypoxic and contain more functional vessels

**Figure 2:**
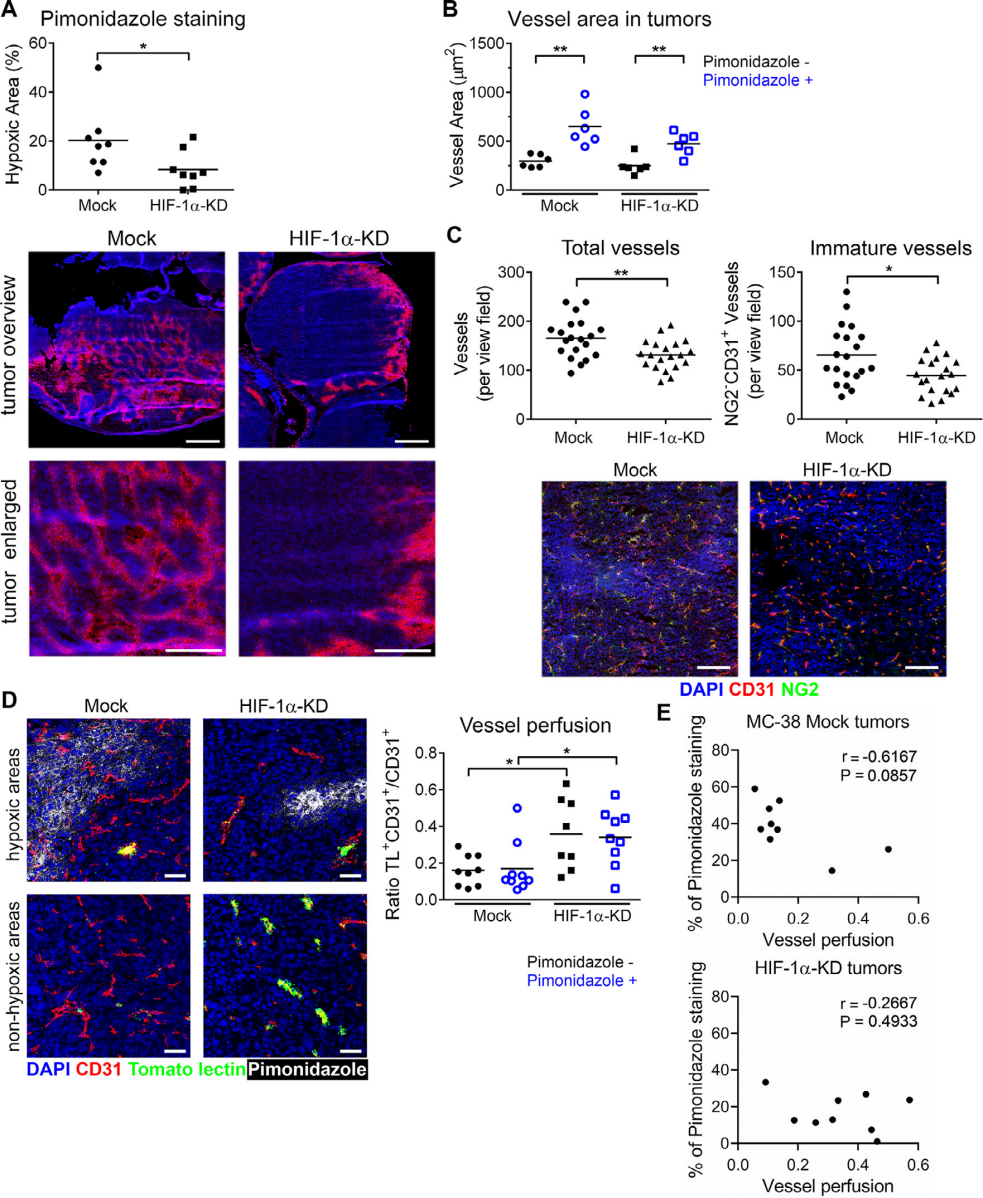
Impaired HIF-1α signaling in MC-38 tumor cells results in vascular normalization in tumors. (A) Quantification of hypoxic regions in tumors as percentage of pimonidazole positive area over total tumor area in Mock and HIF-1α-KD tumors. Representative images of hypoxic regions in cecum tumors visualized by pimonidazole staining, red; counterstained with DAPI, blue (lower panel). Scale bar = 1 mm (overview); scale bar = 500 µm (enlarged) panels. (B) Total vessel area in pimonidazole positive and negative regions of orthotopic tumors determined by counting of CD31-positive vessels. Comparison of Mock and HIF-1α-KD tumors from 3 independent experiments. (C) Total number of vessels (CD31^+^) per view field (PVF) in Mock and HIF-1α-KD tumors (left panel) and immature vessels as determined by NG2-staining of pericytes; NG2^−^CD31^+^; (right panel). Representative immunofluorescence images of tumors stained for NG2 (green), CD31 (red) and DAPI (blue). Three random view fields were counted per tumor, *n* = 4–5 mice per group. Scale bar = 200 µm. (D) Representative images of vessels perfused with tomato lectin (TL; green) in pimonidazole positive (white) and pimonidazole negative tumors regions. Vessels were stained with CD31 Ab (red) and counter-stained with DAPI (blue). Scale bar = 50 µm. Ratio of perfused vessels (TL^+^/CD31^+^) over total number of vessels (CD31^+^) per view field, assessed in both pimonidazole positive and negative regions (right panel). Three random view fields were analyzed per tumor, *n* = 3 per mouse group. Mice from 3 independent experiments were analyzed at day 28 post-tumor cell injection in panels A-C. (E) Correlation analysis between the degree of tumor perfusion and tumor hypoxia (pimonidazole-positive areas) in Mock and HIF-1α-KD tumors, respectively from histological sections shown in panel D. Statistical significance was assessed using the Mann-Whitney test; *, *P*< 0.05; **, *P*< 0.01. (Color version of figure is available online.)

To assess whether the reduced hypoxia response in tumor cells alters hypoxia in HIF-1α-KD tumors, we injected pimonidazole, which forms covalent adducts if the oxygen partial pressure falls below approximately 10 mm Hg. Significant reduction of the hypoxic region was detected in HIF-1α-KD tumors when compared to Mock tumors of similar size (Figure 2A). Next, we analyzed the tumor vasculature and determined the size of blood vessels (vessel area) that allows assessment of angiogenesis. We observed larger vessel areas (CD31^+^) in hypoxic (pimonidazole^+^) regions when compared to nonhypoxic (pimonidazole^−^) regions, which was independent of HIF expression by tumor cells (Figure 2B). This observation is in agreement with the well-known effect of hypoxia on induction of vessel formation [29]. However, HIF-1α-KD tumors showed a reduced intra-tumoral total number of vessels when compared to Mock tumors (Figure 2C), indicating changes in angiogenesis. When we analyzed the vessel maturation by detection of pericytes, using anti-NG2 Ab, a significantly increased number of immature, NG2-negative vessels were detected in Mock tumors when compared to HIF-1α-KD tumors (Figure 2C). The vessel functionality was determined with tomato lectin-FITC perfusion of tumor-bearing mice (Figure 2D). HIF-1α-KD tumors showed more perfused, tomato lectin-positive, vessels. The number of perfused vessel was higher in HIF-1α-KD tumors irrespective of hypoxic and nonhypoxic tumor regions. The correlation between the degree of vessel perfusion and the pimonidazole staining confirmed the increased perfusion in HIF-1α-KD tumors (Figure 2E). To determine how the reduced responsiveness of tumor cells to hypoxia affects tumor angiogenesis, we analyzed tumor lysates using an angiogenesis protein antibody array (Supplementary Figure 2). The analysis of 53 different proteins showed higher amounts of proangiogenic factors (e.g., angiopoetin-1, Cyr61) in Mock tumors when compared to HIF-1α-KD tumors. These data indicate that the impaired hypoxia response in HIF-1α-KD colorectal tumors is associated with reduced vessel formation.

### Alterations of inflammatory and angiogenic genes in orthotopic HIF-1α-KD tumors

**Figure 3:**
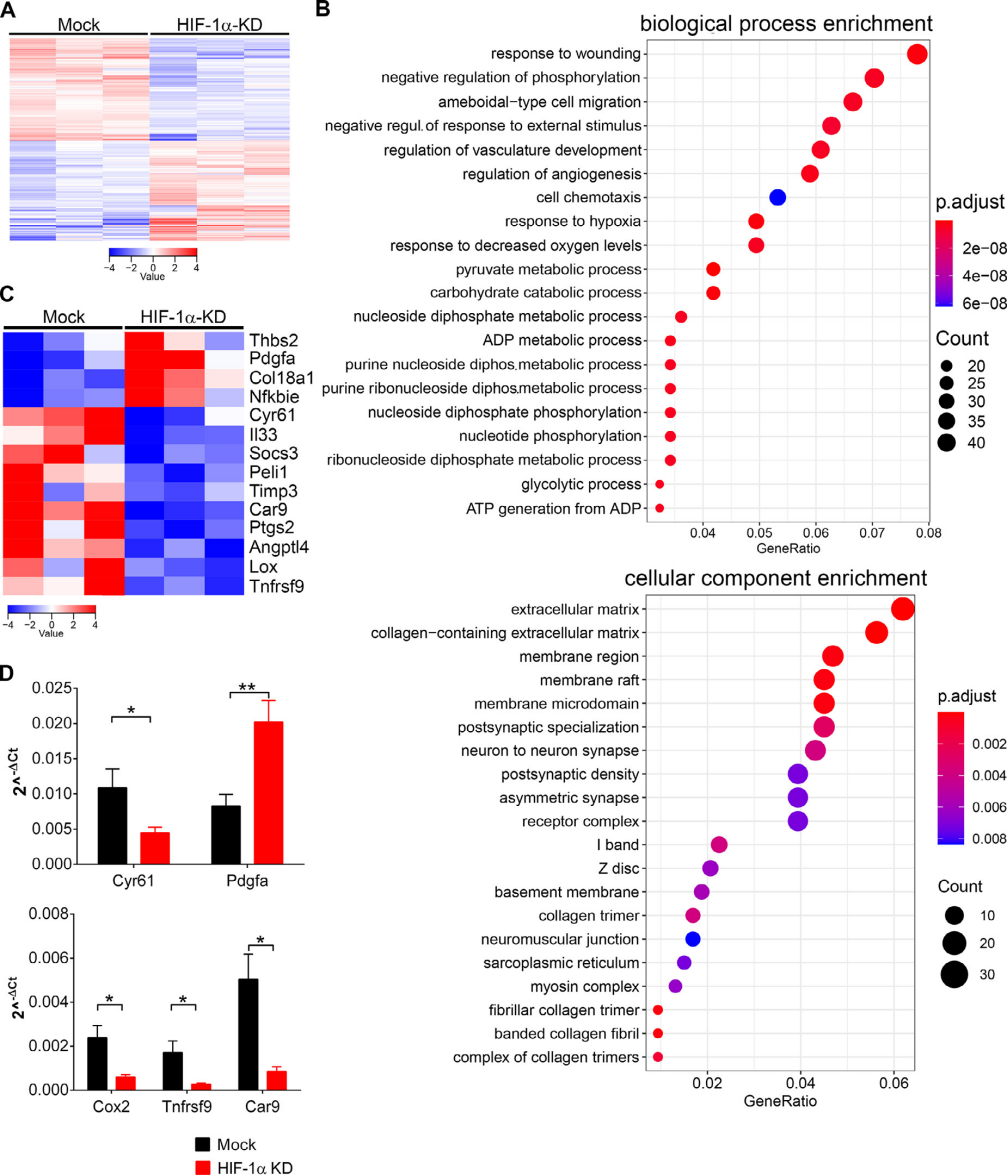
Upregulation of vessel-stabilizing factors and downregulation of proangiogenic molecules in HIF-1α-KD orthotopic tumors. (A) RNA sequencing results from Mock and HIF-KD sorted tumor cells from orthotopic tumors (>50,000 cells) presented as a heatmap. (B) Gene Ontology enrichment analysis performed on genes that were significantly deregulated in sorted HIF-1α-KD when compared to Mock tumor cells. (C) Fourteen genes linked to angiogenesis and inflammation which are differently regulated between HIF-1α-KD and Mock tumor cells. Statistically significant differences: at least *P* < 0.01. (D) Expression levels of selected differentially regulated genes in HIF-1α-KD and Mock tumor cells, normalized to ribosomal protein S12 mRNA, were validated by qPCR (*n* = 4–5). Statistical significance was assessed with Mann-Whitney test; *, *P* < 0.05; **, *P* < 0.01.

To understand the role of HIF-1α on tumor growth, we sorted MC-38GFP tumor cells from orthotopic HIF-1α-KD and Mock tumors and analyzed the transcriptome using RNA sequencing. In HIF-1α-KD tumor cells we found 280 genes significantly upregulated (*P*< 0.05), while 288 genes were downregulated when compared to Mock tumor cells (Figure 3A, Table S1).

Gene ontology enrichment analysis using clusterProfiler_3.16.0 on mouse genome wide annotation revealed an upregulation of genes involved in biological processes and cellular components (Figure 3B). Specifically, regulation of vasculature development and angiogenesis together with cell chemotaxis were increased in HIF-1α-KD tumor cells when compared to Mock tumor cells. Simultaneously, cellular components of extracellular matrix and basement membrane genes were increased. The disease enrichment analysis of identified genes in DisGeNET (a curated human dataset of gene-disease association [30] confirmed the tumor angiogenesis as one of the significantly upregulated pathways (Supplementary Figure 3)). To understand the observed changes in leukocyte infiltration and angiogenesis, we focused on genes involved in these processes. Several genes involved in angiogenesis and vessel maturation, including endostatin gene *Col18a1, Pdgfa Peli1 Angptl4* and *Cyr61*, were found to be differentially regulated (Figure 3C). Interestingly, factors contributing to blood vessel stabilization, such as endostatin derived from collagen XVIII and *Pdgfa*, were upregulated in HIF-1α-KD tumor cells.

Endostatin production depends on the proteolytic cleavage of collagen XVIII by matrix metalloproteinases [31]. Indeed, higher MMP9 expression was detected in lysates of HIF-1α-KD tumors (Supplementary Figure 2), which is in agreement with observed reduced number of blood vessels (Figure 2C). In addition, factors inducing inflammatory responses, like *Peli1, Pgts2, Socs3,* and *Tnfrsf9*, were downregulated, while an inhibitor of the NF-κB pathway (*Nfkbie*) was upregulated in HIF-1α-KD tumor cells. Validation of several identified mRNA species by real-time qPCR confirmed the downregulation of the proangiogenic *Cyr61*and *Pgts2* as well as the proinflammatory *Cox2 and Tnfrsf9* transcripts and the upregulation of a vessel-stabilizing factor *Pdgfa* in HIF-1α-KD tumor cells (Figure 3D). Of note, expression of carbonic anhydrase 9, a typical marker of hypoxia, was also reduced in HIF-1α-KD tumor cells. These data show that the hypoxic response in tumor cells directly induces the release of factors modulating inflammation and angiogenesis within the tumor microenvironment.

Since HIF-1α expression in colorectal cancer correlates with poor prognosis [32,33], we examined human colorectal tumor samples in the Medisapiens database (http://ist.medisapiens.com/) for the expression of HIF-1α and transcripts identified to be dysregulated in HIF-1α-KD tumor cells. The analysis of 991 patients dataset showed a direct correlation between *HIF-1Α* mRNA levels and *LOX, CYR61, PGTS2, SOCS3, TIMP3, PELI1, TNFRSF9,* and *ANGPTL4* (Supplementary Figure 4A), which is in agreement with data obtained from the MC-38 mouse model. Similarly, the analysis using 594 colorectal cancer patient samples from Colorectal Adenocarcinoma dataset (TCGA, PanCancer Atlas; https://www.cbioportal.org/ ver. 3.4.3) confirmed the significant correlation between HIF-1α and the genes identified in MC-38 model (Supplementary Figure 4B).

### Intratumoral delivery of CYR61 increases tumor hypoxia and vascular permeability in HIF-1α-KD tumors

**Figure 4:**
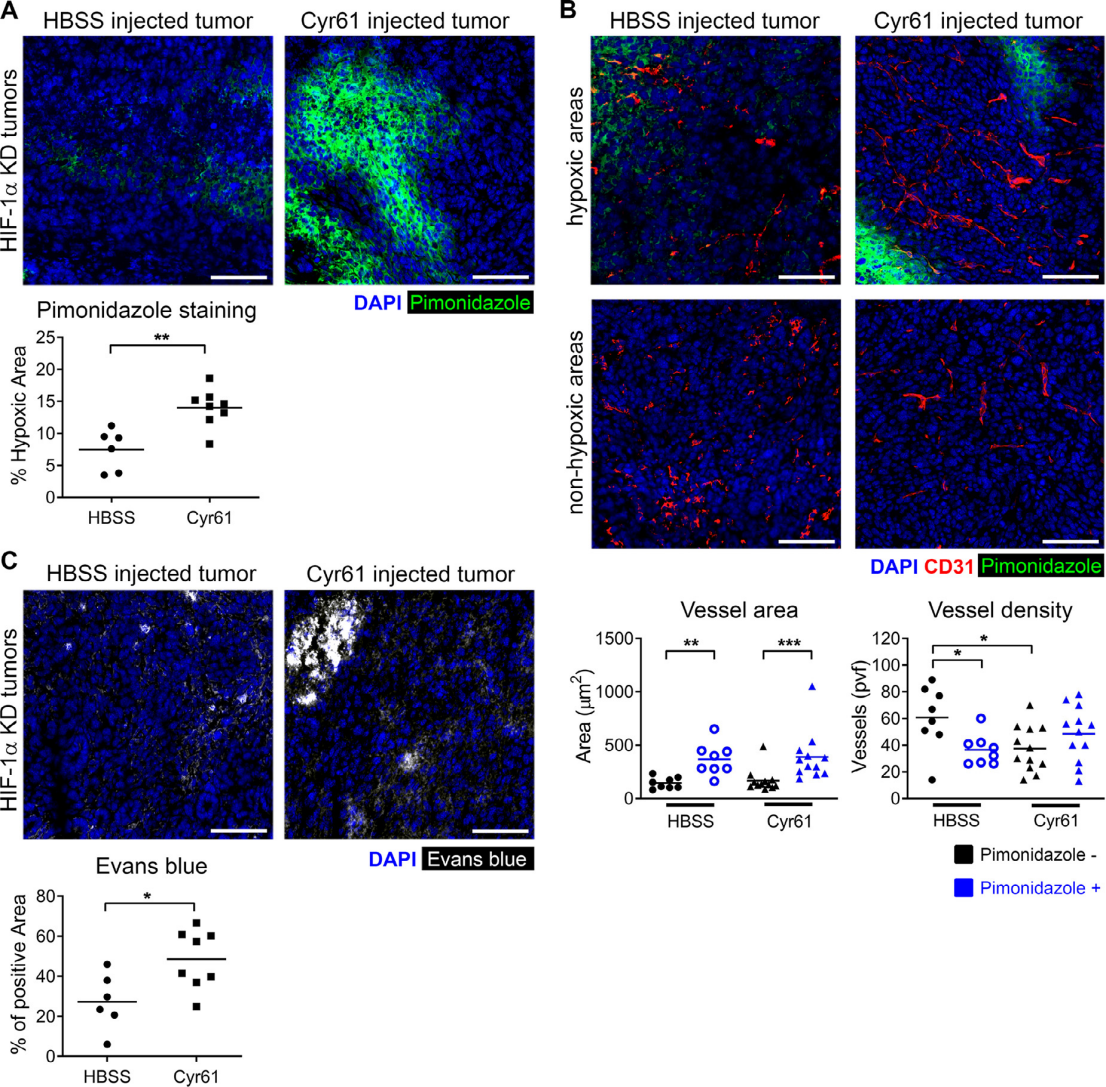
Intratumoral treatment of HIF-1α-KD orthotopic tumors with Cyr61 protein increases tumor hypoxia and vascular leakiness. (A) Representative images of hypoxic regions in HIF-1α-KD tumors treated with Cyr61 or HBSS (control) visualized by pimonidazole staining (green) and counterstained with DAPI (blue). Quantification of hypoxic regions as percentage of pimonidazole positive area over total tumor area in respective tumors (lower panel); n = 6–8 tumors per group. (B) Representative images of blood vessels (CD31 staining, red) in HIF-1α-KD tumors either treated with Cyr61 or HBSS. Hypoxic and non-hypoxic regions visualized by pimonidazole (green) and counterstained with DAPI (blue) for respective treatments. Lower panels show total vessel area (CD31^+^) and the average vessel density per view field (PVF), determined in randomly chosen 2 view fields per tumor sample (*n* = 4–5 tumors per group). (C) Representative images of Evans Blue staining (white) in HIF-1α-KD tumors treated with either Cyr61 or HBSS. Quantification of vascular permeability in tumors shown as a percentage of Evans Blue positive area over total tumor area (*n* = 5–8 tumors per group). Statistical significance was assessed using the Mann-Whitney test; *, *P*< 0.05; **, *P*< 0.01; ***, *P*< 0.001. (Color version of figure is available online.)

Pathologic tumor neovascularization and aggressiveness have been linked to the expression of Cyr61/CCN1 in breast and pancreatic cancer cells [34], [35], [36]. Cyr61 is an extracellular matrix protein promoting endothelial cell proliferation, adhesion, and differentiation [37]. We observed downregulation of Cyr61 in HIF-1α-KD tumor cells (Figure 3C-D) as well as in tumors (Supplementary Figure 2). To test whether Cyr61 affects the tumor growth of colon carcinoma, we intratumorally injected recombinant Cyr61 into HIF-1α-KD tumors. Increased hypoxic regions, determined by pimonidazole staining, were observed upon Cyr61 injection when compared to control tumors treated with the solvent HBSS (Figure 4A). No changes in vessel density were observed between Cyr61 and control tumors in the hypoxic regions while a significant reduction of vessel numbers was observed in pimonidazole-negative regions (Figure 4B). However, Cyr61 injection induced larger vessels, as determined by the vessel area, which correlated with reduced vessel density in pimonidazole-negative regions. To test whether Cyr61 injection affected the functionality of vessels, we injected Evans Blue and assessed extravasation of the dye (Figure 4C). HIF-1α-KD tumors injected with recombinant Cyr61 showed increased vascular permeability, which is in agreement with Cyr61 as an inducer of aberrant tumor angiogenesis associated with nonfunctional leaky vessels and hypoxia.

### HIF-1α interacts with TRAF6 upstream of NF-κb in MC-38 cells

**Figure 5:**
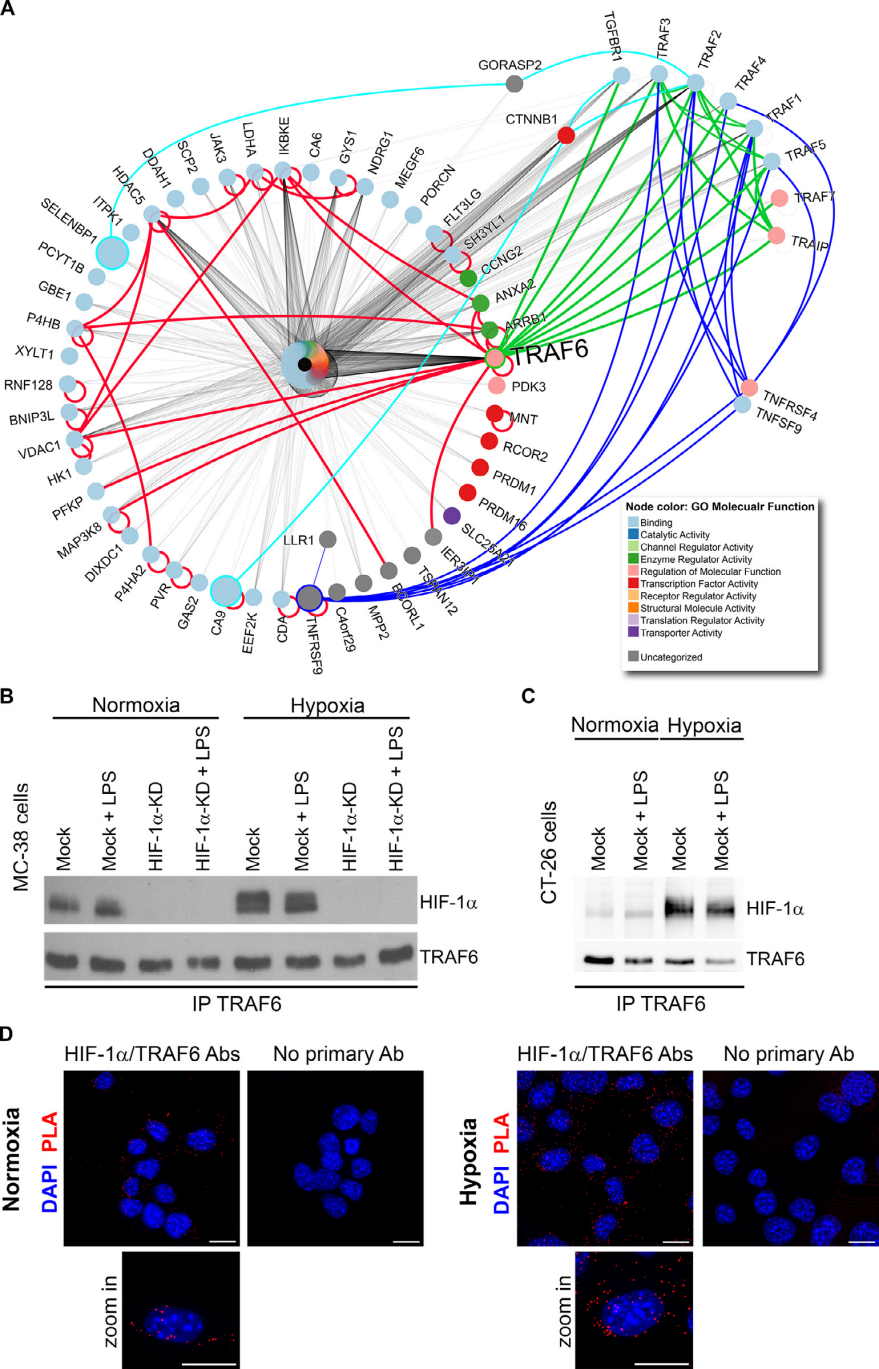
HIF-1α interacts with TRAF6 and links hypoxia to inflammation in colorectal cancer. (A) Protein-protein interaction network based on differently regulated genes observed in MC-38 cells cultured under either hypoxia or normoxia using the NAViGaTOR software. Node size is proportional to the expression level in hypoxia versus normoxia. Red lines represent direct interactions among proteins upregulated during hypoxia. Blue, turquoise, and green lines show indirect/mediated interactions of the TRAF6 with other hypoxia upregulated proteins. (B) Coimmunoprecipitation of TRAF6 and HIF-1α in Mock and HIF-1α-KD MC-38 cells stimulated either by hypoxia/normoxia with or without addition of LPS followed by the detection of TRAF6 or HIF-1α, respectively (n = 3). (C) Co-immunoprecipitation of TRAF6 and HIF-1α in CT26 cells stimulated either by hypoxia/normoxia or LPS addition, followed by the detection of TRAF6 or HIF-1α, (*n* = 3). (D) Proximity ligation assay in MC-38 Mock cells cultured under normoxia and hypoxia for 8 h followed by detection with HIF-1α and TRAF6-specific antibodies using Duolink In Situ Orange Kit (Sigma). Negative control (No primary Ab) is shown; scale bar = 15 µm. (Color version of figure is available online.)

Inflammation is directly linked to colon cancer development [12] and the colonic epithelium is known to be in a physiologically hypoxic state [18]. This unique physiological situation in the colon stimulated us to assess the cross talk between inflammation and hypoxia in the mouse colon-cancer model. Previously, we had shown that HIF-1α levels in MC-38 cells remain unchanged upon LPS stimulation, while hypoxia treatment failed to induce NF-κB signaling in vitro [19]. We used this dataset of differently expressed genes from MC-38 cells for an *in silico* analysis based on the known proteome-wide physical protein-protein interactions (Figure 5A). Of note, the HIF target and hypoxic tumor hallmark gene carbonic anhydrase 9 (CA9) was identified among the upregulated genes, which confirms the hypoxic culture conditions. Among the identified sixty proteins, the tumor necrosis factor receptor-associated factor 6 (TRAF6) protein was identified as a possible key interaction point with the highest centrality value between the hypoxic and inflammatory pathways (Figure 5A). Previously, TRAF6 overexpression in tumor cells had been shown to stabilize HIF-1α [38]. Thus, we tested the hypothesis that TRAF6 directly interacts with HIF-1α, as suggested from the protein-protein interaction network analysis. HIF-1α-KD and Mock MC-38 cells were cultured under normoxia and hypoxia, with or without LPS stimulation, followed by TRAF-6 immunoprecipitation and HIF-1α detection (Figure 5B). Increased co-immunoprecipitated HIF-1α protein amounts were detected in Mock cells under hypoxia when compared to normoxia, indicating physical interaction between TRAF6 and HIF-1α. When HIF-1α was immunoprecipitated in MC-38 cells stimulated with hypoxia, TRAF6 protein was detected (Supplementary Figure 5). LPS stimulation alone had no effect on HIF-1α expression (Figure 5B), which is in agreement with our previous data [19]. To demonstrate that the HIF-1α-TRAF6 interaction is not cell type specific, coimmunoprecipitation was confirmed in murine CT26 colon carcinoma cells (Figure 5C). Next, we confirmed the TRAF6-HIF-1α interaction using proximity ligation assay in situ. MC-38 Mock cells under normoxia showed interaction between TRAF6 and HIF-1α in the cytoplasm, which was increased in cells cultured under hypoxia (Figure 5D).

### TRAF6 is required for orthotopic tumor growth

**Figure 6:**
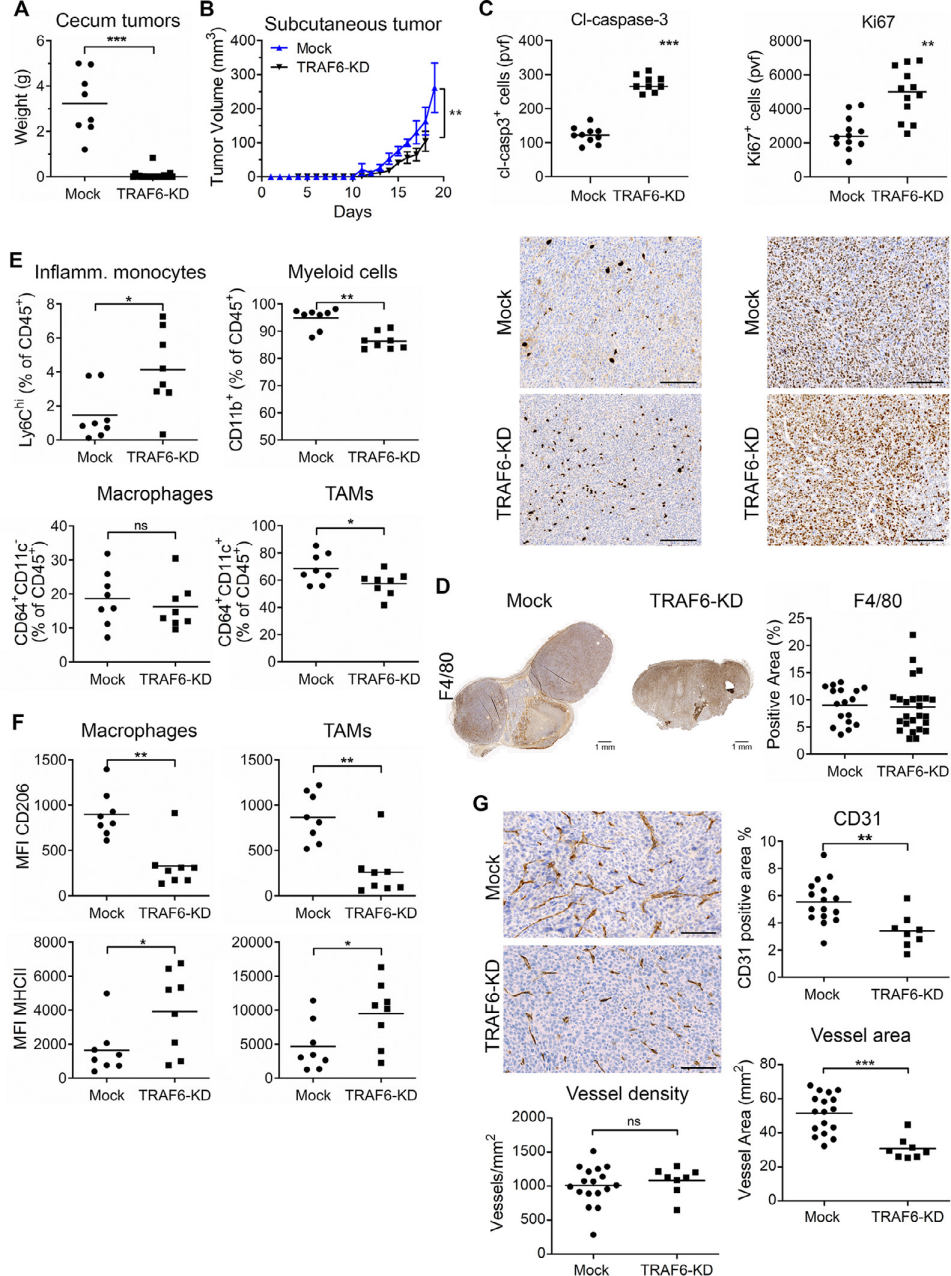
TRAF6-KD in MC-38 cells impairs tumor growth in the cecum and reduced subcutaneous tumor growth with changes in macrophages and angiogenesis. (A) Tumor mass in mice with MC-38 TRAF6-KD and Mock cell injection in the cecum 28 days postinjection. Mice without tumor have zero value. Mice from 2 independent experiments are presented (TRAF6-KD; *n* = 21). (B) The growth of subcutaneous tumors in mice injected either with TRAF6-KD or Mock MC-38 cells (n ≥ 10 per group). (C) Immunohistochemical analysis of cleaved-caspase-3 (cl-casp-3) and Ki67 staining in subcutaneous MC-38 Mock and TRAF6-KD tumors. Random view fields were quantified for positive cells. pvf, per view field; scale bar = 200 µm. (D) Immunohistochemical analysis of subcutaneous tumors stained with the macrophage marker F4/80 Ab (top panels) were quantified (low panel). Three to 4 random view fields were analyzed per tumor; *n* = 4–5 per group. (E) Number of innate immune cells in orthotopic tumors represented as percentage of living CD45^+^ cells, determined by flow cytometry; inflammatory monocytes, Ly6C^hi^; dendritic cells, CD103^+^/CD64^−^; macrophages, (CD64^+^/CD11c^−^/CD103^−^; and tumor-associated macrophages – TAMs, CD64^+^/CD11c^+^/CD103^−^. Tumor analysis from two independent experiments (*n* = 8). (F) Cell surface expression of polarization markers MHCII and CD206 was analyzed by flow cytometry, in parallel to the experiments shown under E. MFI from FMO control was subtracted from each sample; MFI, median fluorescence intensity. (G) Angiogenesis in TRAF6-KD and Mock tumors was analyzed using CD31 staining. The amount to vessel (CD31-positive staining) was quantified as percentage of total tumor area; vessel area and vessel density, respectively. Representative images of Mock and TRAF6-KD tumor are shown; scale bar = 100 µm. Statistical significance was assessed using the Mann-Whitney test; *, *P*< 0.05; **, *P* < 0.01; ***, *P* < 0.001.

To test the involvement of TRAF6 in tumorigenesis, we prepared MC-38GFP with shRNA-induced downregulated TRAF6 expression, MC-38-TRAF6-KD (Supplementary Figure 6A-C), and injected them into the cecum wall. Interestingly, only three out of twenty one animals developed a very small tumor upon MC-38-TRAF6-KD tumor cells injection while all Mock cell injections led to tumor formation (Figure 6A). To exclude that TRAF6-KD tumor cells have a general problem to proliferate in vivo, we intravenously injected these cells and followed metastasis to the lungs. Lung tumors grew in all mice injected with MC-38-TRAF6-KD cells at similar size as with Mock cells, albeit in reduced numbers (Supplementary Figure 6D). Subcutaneous injection of MC-38-TRAF6-KD tumor cells also resulted in normal tumor development, albeit the size of tumors was reduced when compared to Mock tumors (Figure 6B). These data indicate that TRAF6 in colorectal tumor cells is essential for tumor growth specifically in the colon/cecum.

Next, we analyzed the composition of TRAF6-KD and Mock subcutaneous tumors. TRAF6-KD tumors showed an increased detection of apoptotic cells, as determined by cleaved-caspase 3 staining, when compared to Mock tumors, which correlates with reduced tumor size (Figure 6C). Interestingly, TRAF6-KD tumor showed also increased number of Ki67-positive tumor cells. No major changes in macrophages between TRAF6-KD and Mock tumors were observed using immunohistochemistry (Figure 6D). Flow cytometry analysis of tumor infiltrating leukocytes revealed an increased presence of inflammatory monocytes (Ly6C^hi^), and reduced numbers of myeloid cells (CD11b^+^) and tumor-associated macrophages; TAMs (CD11b^+^CD64^+^CD11c^+^) in TRAF6-KD tumors (Figure 6D). The number of macrophages (CD64^+^CD11c^−^) remained unchanged between TRAF6-KD and Mock tumors. When we analyzed the polarization of macrophages into protumorigenic M2 or antitumorigenic M1 one using the CD206 and MHCII markers, respectively, increased numbers of M1 type macrophages in TRAF6-KD were observed (Figure 6E), which is in agreement with the observed tumor size reduction (Figure 6B).

### TRAF6-KD tumors show reduced vessel density

TRAF6 had previously been shown to modulate HIF-1α expression in colonic and breast cancer tumor cells [38,39], and here we showed that TRAF6 interacts with HIF-1α in murine MC-38 and CT-26 cells. Thus, we asked whether TRAF6-KD affected tumor angiogenesis. While the number of vessels was not affected in TRAF6-KD tumors, a significantly reduced vessel area, reflecting reduced angiogenesis, was observed when compared to Mock tumors (Figure 6F). These results indicate that that functional TRAF6 signaling contributes to formation of aberrant blood vessels in the tumors, reflected in significantly larger vessels.

## Discussion

Inflammation and hypoxia modulate the tumor microenvironment to promote tumor progression and invasiveness [40]. Low oxygen tensions, present at both inflammatory sites and tumors, stabilize HIF-αs, which activates target genes involved in angiogenesis, cell survival, metabolic reprogramming, and metastasis. Overexpression of HIF-1α was observed in various human cancers, including colorectal cancer [41] and is independently associated with poor prognosis [32]. HIF-1α is a transcription factor regulating tumor cell survival and proliferation; and epithelial barrier function following inflammation in the colon [11,^42^,43]. Here we studied the role of HIF-1α-mediated response towards hypoxia in an orthotopic colorectal cancer model in immunocompetent mice. Importantly, we observed smaller HIF-1α-KD tumors, with reduced infiltration by granulocytes. The almost complete absence of a hypoxic response in MC-38 cells resulted in tumors with less but more functionally perfused vasculature. The number and the size of blood vessels in Mock tumors correlated with increased tumor hypoxia indicating an active role of tumor cell-derived HIF-1α signaling leading to aberrant, non-functional angiogenesis [3]. In this context, down-regulation of HIF-1α in tumor cells resulted in vessel normalization and maturation, and consequently less hypoxia. Of note, increased local oxygen consumption by infiltrating granulocytes with active respiratory burst contributes to intestinal tissue hypoxia in colitis models [44]. Indeed, we observed higher numbers of granulocytes in Mock tumors. Recently, metabolic targeting of HIF-1α has been shown to potentiate chemotherapy responses in human colorectal cancer tumor growth in a murine model, which was associated with reduced angiogenesis [45]. These findings are in agreement with our data showing that MC-38 tumors with reduced HIF-1α expression are smaller, with functional vessels and reduced hypoxia. In addition, better-perfused vasculature may explain the increased responsiveness to chemotherapy observed previously [45]. Recent study provided evidence for microRNA regulated switch between endothelial cell proliferation and migration during angiogenesis [46]. Specifically, miRNA-29a-3p was found to be a selective marker identifying colorectal cancer patients, who are responding to anti-angiogenic treatments. Using mirDIP database portal we identified possible hsa-miR-29a-3p target genes in our tumor dataset. Interestingly, 81 targets overlap with upregulated genes and 87 targets with down-regulated genes (Supplementary Figure 7); including upregulated genes involved extracellular matrix modification and downregulated expression of Cyr61. Further studies are required to assess the role of the tumor hypoxia status and its effect on miRNA regulated angiogenesis.

The balance between pro- and anti-angiogenic factors is usually disturbed during tumor development, resulting in tortuous and leaky vessels [47]. Analysis of tumor lysates showed reduced amounts of inflammatory and pro-angiogenic factors in HIF-1α-KD tumors, such as Cyr61 or Dll4, which correlated with a normalized vasculature. Transcriptome analysis of sorted MC-38 tumor cells revealed upregulation of vessel-stabilizing factors such as endostatin, Pdgfa, Thbs2 in HIF-1α-KD cells and reduced expression of pro-angiogenic factors such as Cyr61, Cox-2 and Angptl4 when compared to Mock tumors. Both findings on mRNA and protein levels are in agreement with histological evidence of more functional vessels detected in tumors with an impaired hypoxia response. Of note, HIF-1α down-regulation resulted in lower transcript levels of genes such as *Pellino1, Socs3* and *Tnfrsf9* involved in the inflammatory cascade. In human colorectal tumors, HIF-1α expression positively correlated with genes shown to be regulated by HIF-1α in MC-38 tumors. Particularly, the observed Cyr61 mRNA level was previously reported to be a pro-angiogenic and tumor-promoting factor in renal, breast, pancreas and lung cancer cell lines [48], [49], [50]. We showed that intra-tumoral injection of Cyr61 resulted in enhanced angiogenesis associated with increased leaky vasculature and tumor hypoxia. Thus, the interference with the HIF-1α response in tumor cells leads to vessel normalization despite the intact hypoxia response in the tumor microenvironment.

Tumor cell-derived NF-κB signaling is required for tumor initiation and progression in colorectal cancer [17,51]. Specific inactivation of the NF-κB pathway in epithelial cells attenuated the formation of colitis-associated colon cancer [52]. The modulation of NF-κB signaling in colon cancer has been linked to: increased chemokine expression associated with leukocyte infiltration and the microbiota-derived activation [53], [54], [55]. Bioinformatics analysis of the potential HIF-1α interactome identified TRAF6, an upstream mediator of the NF-κB pathway, as a potential link between hypoxia signaling and the inflammatory cascade. MC-38 tumor cells with reduced TRAF6 expression could not establish a tumor in the caecum, while tumors readily formed upon subcutaneous injection, albeit at a reduced size. Intestinal epithelial cells are in a constant contact with microbiota, which requires a tight control of inflammatory responses [54,56], likely explaining why tumor cells with reduced TRAF6 activity and therefore reduced NF-κB signaling do not grow in the inflammatory environment of a colon.

The histological analysis of TRAF6-KD tumors showed increased apoptosis (cleaved-caspase-3^+^), but also increased tumor cell proliferation as determined by Ki67 staining, when compared to Mock tumors. Ki67-staining is routinely being used for detection of proliferating tumors cells in animal models (e.g. [57]. However, recent study showed that Ki67 expression is not always associated with cell proliferation, since the Ki67 gene controls heterochromatin organization [58]. Targeted depletion of Ki67 gene in the mouse gut epithelium did not result in any cell proliferation deficiency. Furthermore, cleaved-caspase 3 detection is a better prognostic marker than Ki67 staining in colorectal and endometrial cancers [59,60]. Recently, suppression of TRAF6 expression through overexpression of miR-146a resulted in deregulation of tumor cell proliferation in non-small cell lung cancer [61]. This finding is in agreement with our observation that TRAF6-KD tumors showed enhanced Ki67 staining, although we observed limited tumor growth due to enhanced apoptosis and reduced angiogenesis.

TRAF6 is a unique member of the TRAF family of intracellular proteins, and is critically involved in the IL-1 receptor and Toll-like receptor family induced signal transduction leading to NF-κB activation [62]. Increased TRAF6 expression has been reported in various tumors, including colon, breast and melanoma cancers, as well as in lymphoid malignancies [38,^39^,[63], [64], [65]. Reportedly, TRAF6 is a K63-E3 ubiquitin ligase that ubiquitinates and stabilizes HIF-1α in colon cancer cells independent of oxygen [38]. On contrary to previous findings, we observed enhanced endogenous levels of HIF-1α interacting with TRAF6 under hypoxia. In breast cancer cells, TRAF6 overexpression correlated with increased HIF-1α signaling and metastasis [39]. We identified TRAF6 as a potential binding partner of HIF-1α in MC-38 tumor cells under hypoxia and confirmed that TRAF6 physically interacts with HIF-1α as visualized by proximity ligation assay. Interestingly, we observed mostly cytoplasmic localization of TRAF6-HIF-1α interactions. Our data indicate that TRAF6-HIF-1α interaction contributes to tumor growth, primarily through modulation of angiogenesis, likely through stabilization of HIF-1α proteins levels in tumor cells [38]. Both individual downregulations of either HIF-1α or TRAF6 resulted in smaller tumors with reduced angiogenesis and normalized vasculature. Thus, targeting of hypoxia-inflammatory crosstalk represents an attractive approach for further development of improved delivery of standard or targeted cancer therapies.

## Authors' contributions

Conception and design: J.F. Glaus Garzon, R.H. Wenger, M.O. Hottiger, L. Borsig; Development of methodology: J. F. Glaus Garzon, L. Borsig; Acquisition of data/Investigation: J.F. Glaus Garzon; Analysis and validation of data (e.g., statistical analysis, biostatistics, computational analysis): J.F. Glaus Garzon, C. Pastrello, I. Jurisica, L. Borsig; Data curation, visualization: C. Pastrello, I. Jurisica; Resources: M.O. Hottiger, R.H. Wenger, I. Jurisica, L. Borsig; Writing original draft: J.F. Glaus Garzon, L. Borsig; Writing/review/editing of the manuscript: J.F. Glaus Garzon, I. Jurisica, R.H. Wenger, M.O. Hottiger, L. Borsig; Study supervision: L. Borsig.

## Appendix Supplementary materials

Image, application 1
