# Supplementary material for: Tumor cell endogenous HIF-1α activity induces aberrant angiogenesis and interacts with TRAF6 pathway required for colorectal cancer development
Source: Neoplasia. 2020 Oct 24;22(12):745–58. doi: 10.1016/j.neo.2020.10.006 (PMC7588814; doi:10.1016/j.neo.2020.10.006)
Supplement: Supplementary file 1 [file mmc1.pdf]

Supplementary Data

Glaus Garzon et al.

Tumor cell endogenous HIF-1α activity induces aberrant angiogenesis and interacts with TRAF6 pathway required for colorectal cancer development

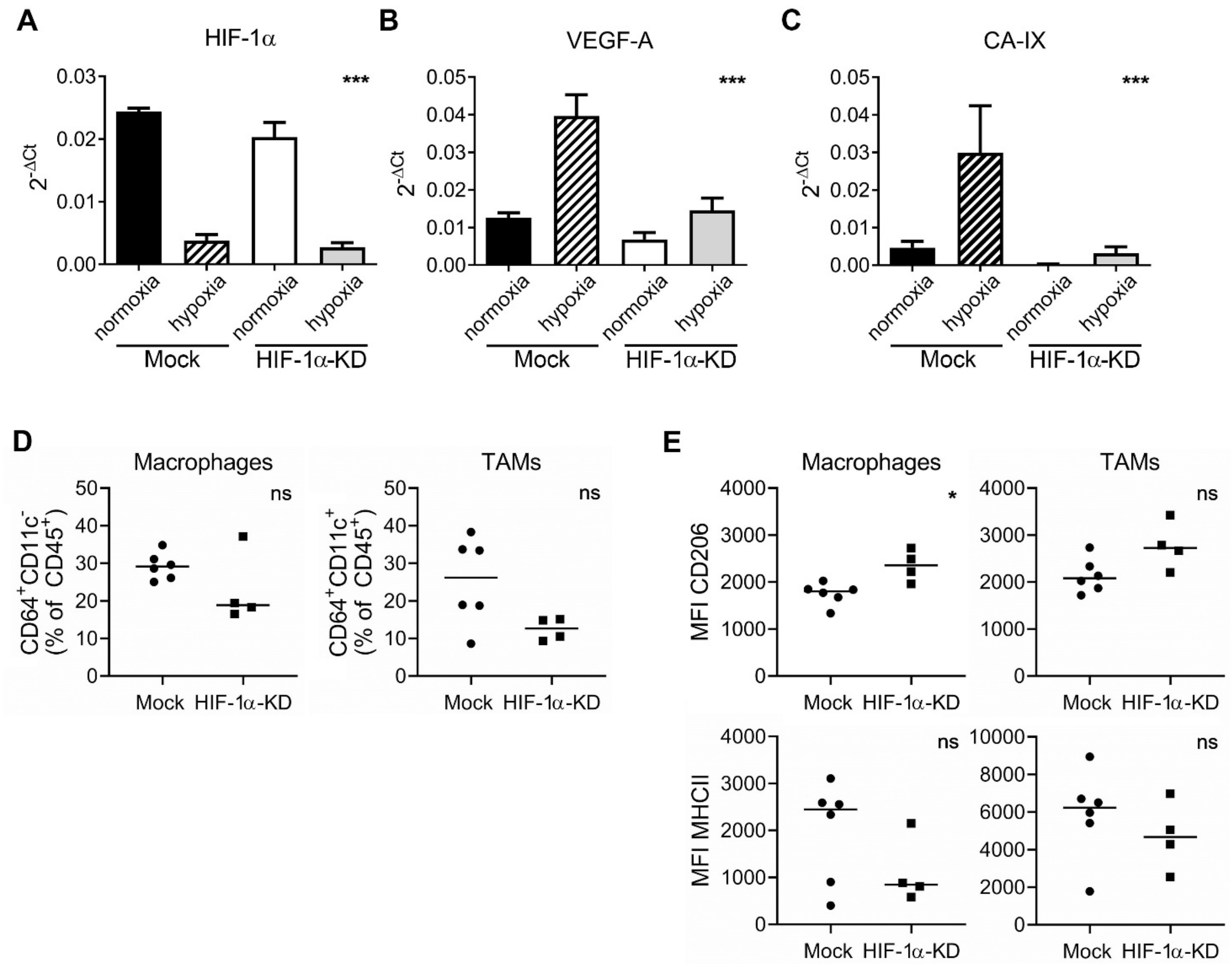

Supplementary Figure 1. Characterization of HIF-1α-KD MC-38 cells. A) Transcript levels of HIF-1α were determined by qPCR after 8 hours incubation under normoxia (21% O_2) or hypoxia (0.2% O_2) stimulation. The expression levels of canonical HIF target genes VEGF-A **(B)** and CA-IX **(C)** were quantified by RT-qPCR after 8 hours incubation under normoxia (21% O_2) or hypoxia (0.2% O_2) compared to mock controls. Expression levels were normalized to constitutively expressed ribosomal protein S12 mRNA levels. **(D)** Number of macrophages ($CD64^+/CD11c^-$) and tumor-associated macrophages - TAMs ($CD64^+/CD11c^+$) in orthotopic tumors represented as percentage of living $CD45^+$ cells, determined by flow cytometry. (**E)** Cell surface expression of polarization markers MHCII and CD206 was analyzed by flow cytometry, in parallel to the experiments shown under D. MFI from FMO control was subtracted from each sample; MFI, median fluorescence intensity. Statistical significance was assessed using the Mann-Whitney test;). *, $p < 0.05$; ***, $p < 0.001$.

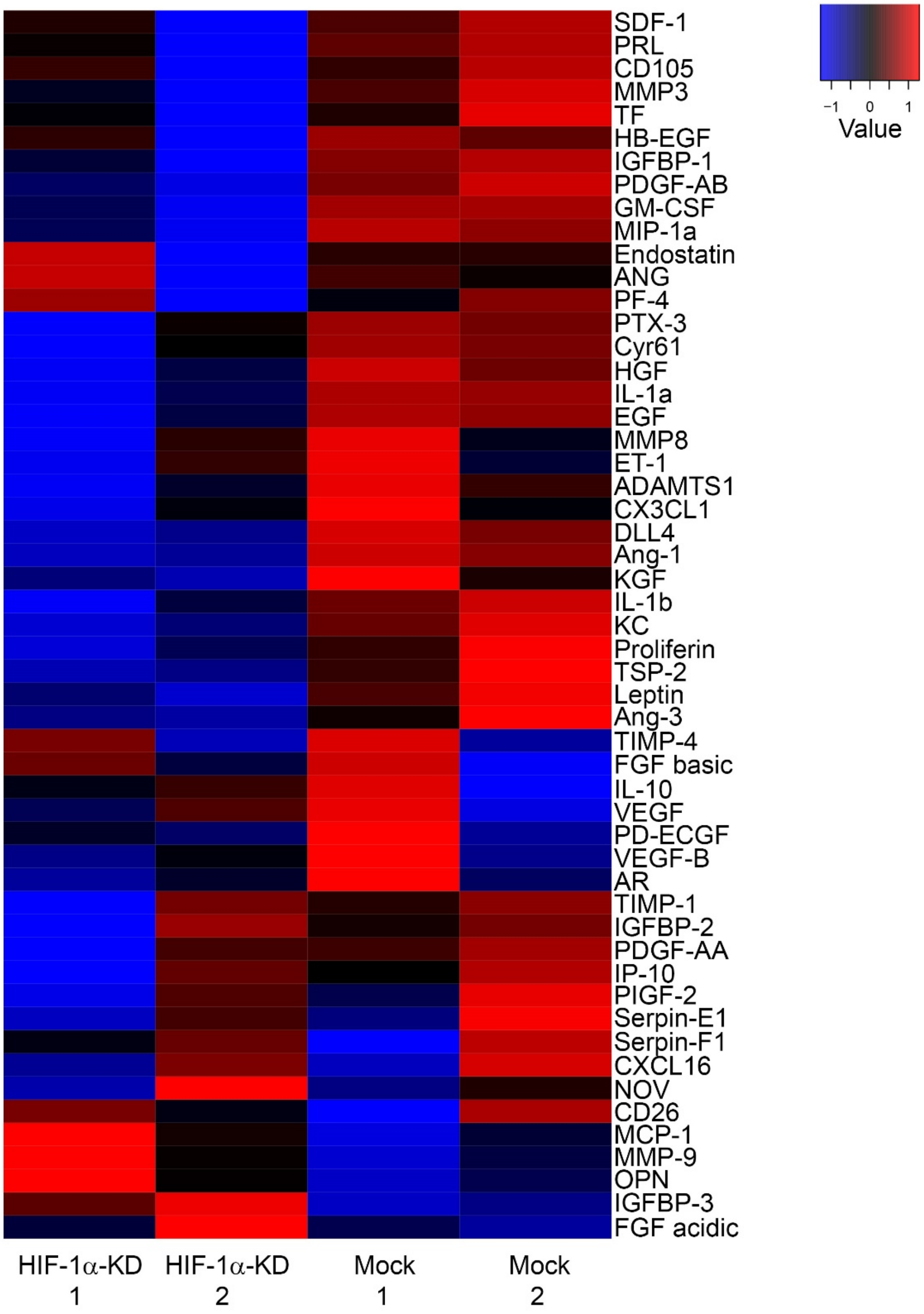

Supplementary Figure 2. Angiogenesis protein arrays of tumor lysates from HIF-1α KD and Mock cecal tumors. Total tumor lysates incubated on Angiogenesis Array. Dot blot pixel densities were quantified and normalized to internal manufacturer's control and plotted as heatmap using web application from Functional Genomic Center Zurich.

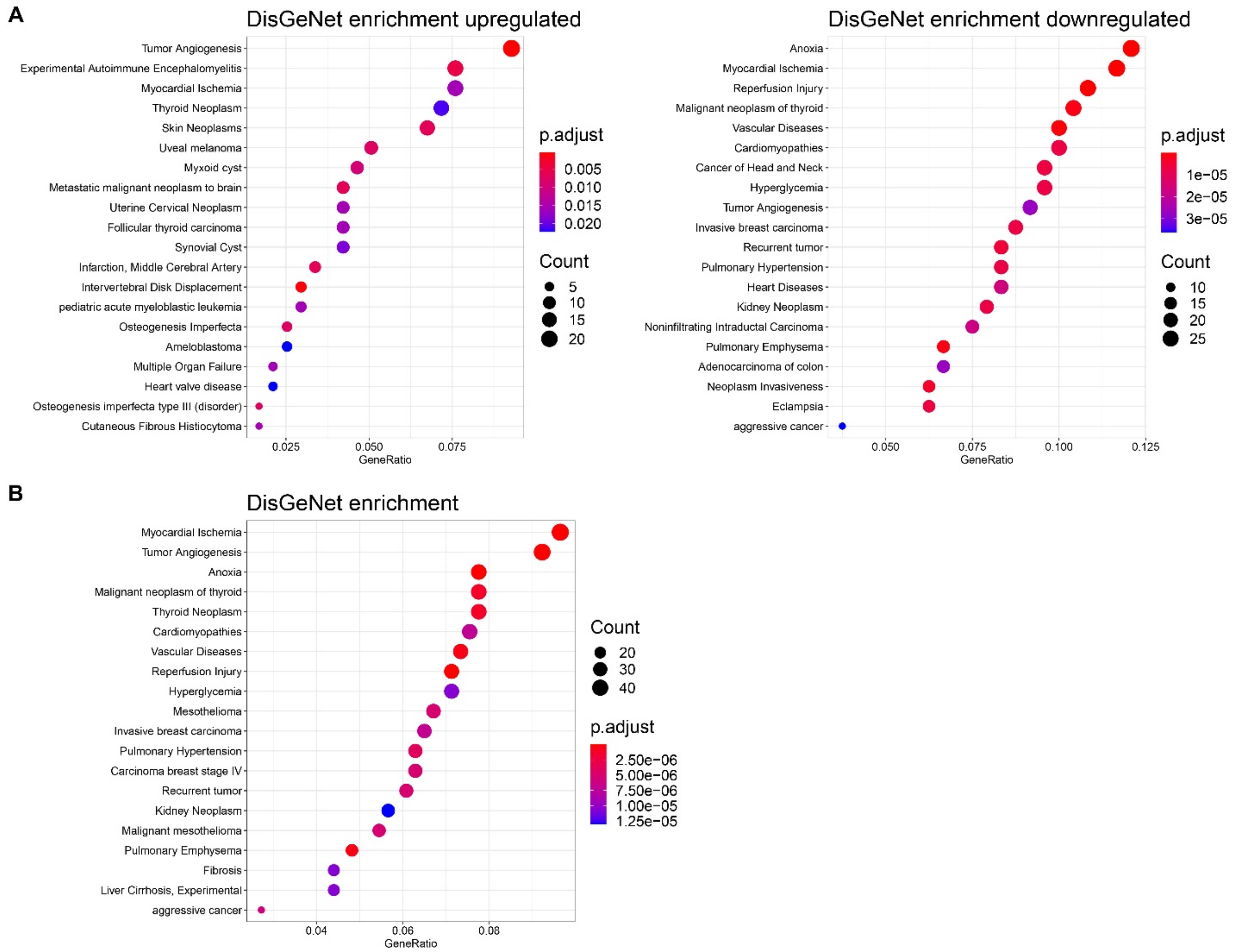

Supplementary Figure 3. Disease enrichment analysis of genes using human curated sets. A) List of genes from MC-38 HIF-1a KD cells versus Mock cells sorted from intracecal tumors were converted to human symbols and used for disease enrichment analysis using DOSE_3.14.0, using DisGeNet (DGN) dataset. Plots were obtained using clusterProfiler

A

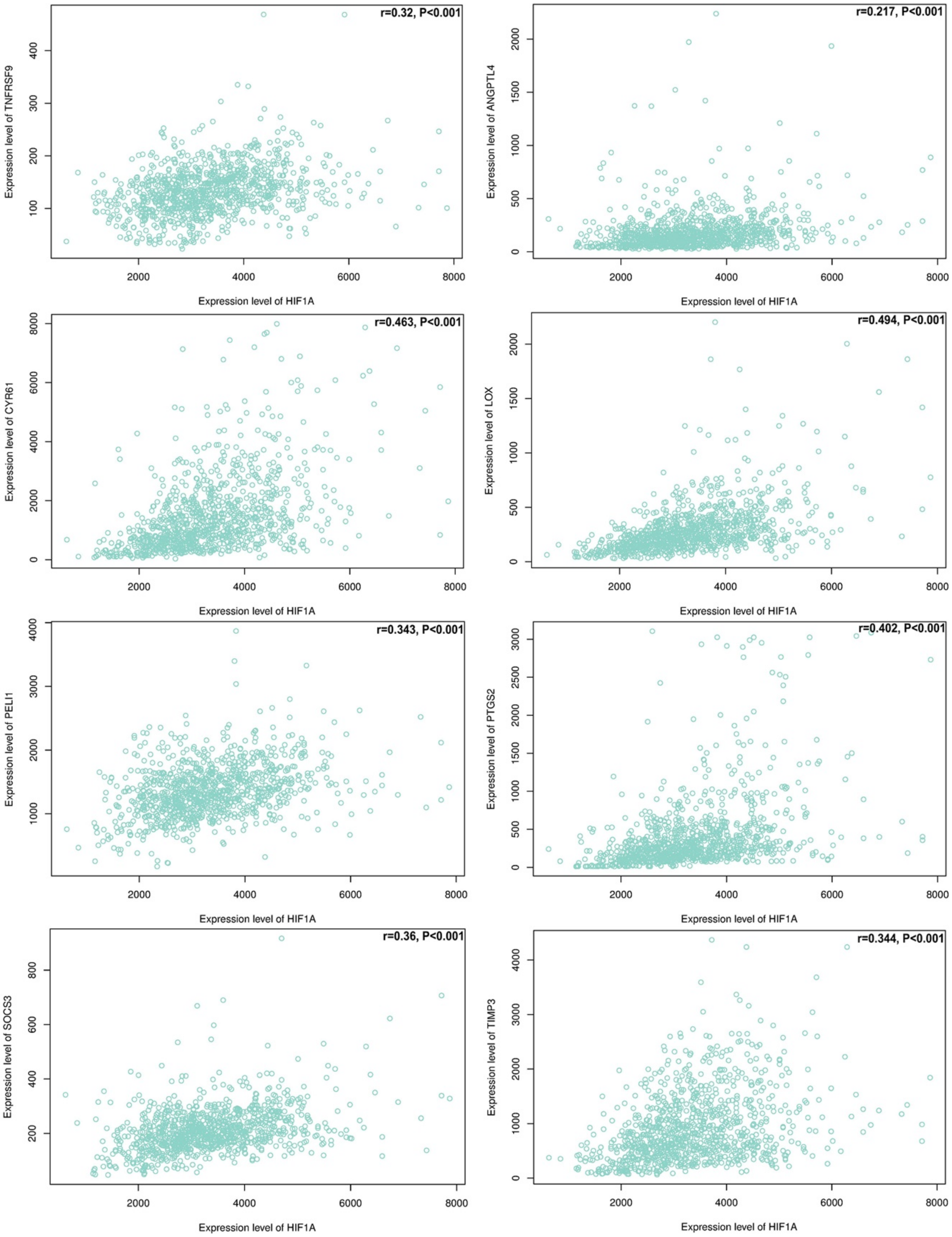
r=0.32, P<0.001
Expression level of TNFRSF9
Expression level of HIF1A
r=0.217, P<0.001
Expression level of ANGPTL4
Expression level of HIF1A
r=0.463, P<0.001
Expression level of CYR61
Expression level of HIF1A
r=0.494, P<0.001
Expression level of LOX
Expression level of HIF1A
r=0.343, P<0.001
Expression level of PELI1
Expression level of HIF1A
r=0.402, P<0.001
Expression level of PTGS2
Expression level of HIF1A
r=0.36, P<0.001
Expression level of SOCS3
Expression level of HIF1A
r=0.344, P<0.001
Expression level of TIMP3
Expression level of HIF1A

B

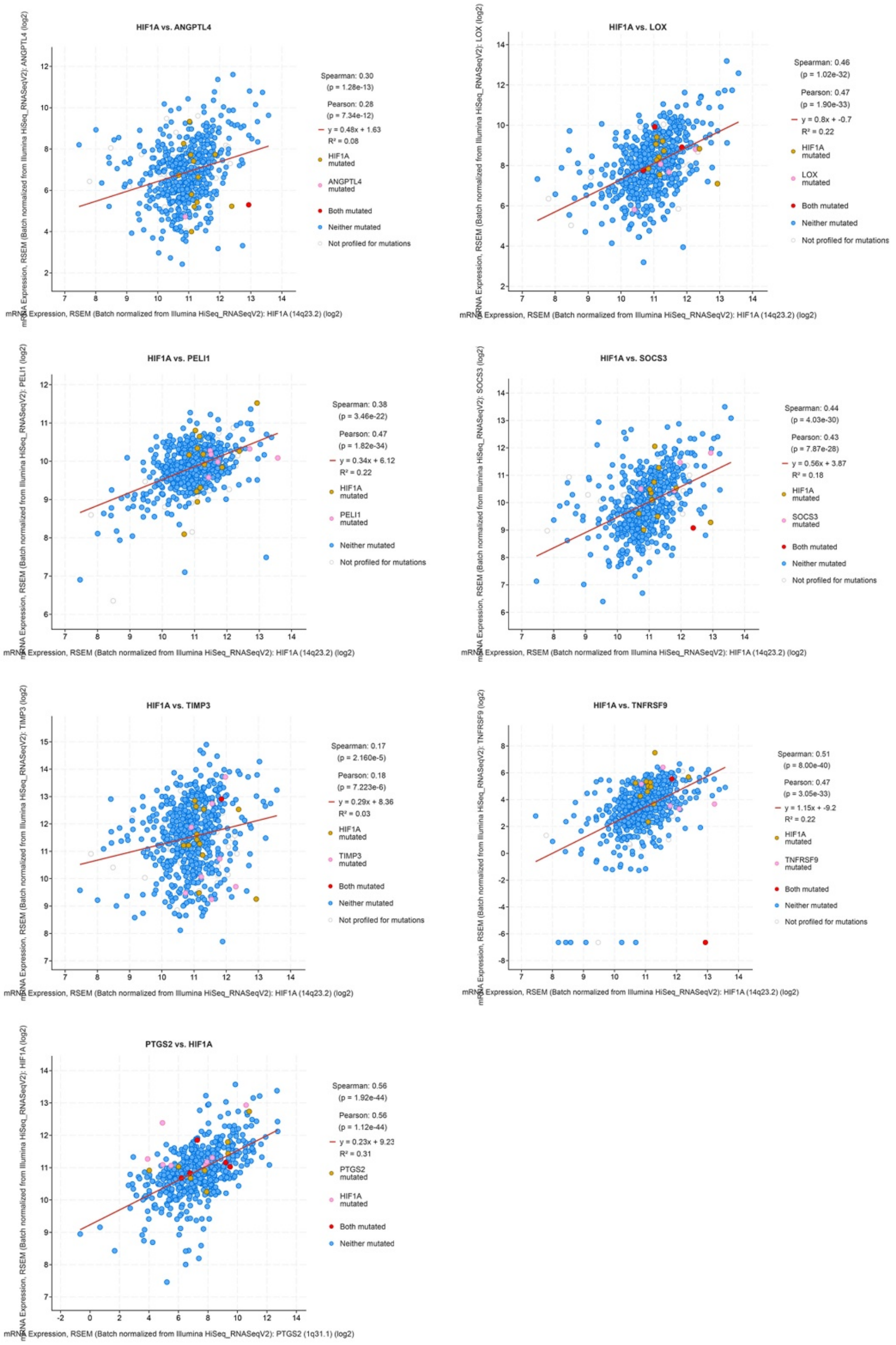

Supplementary Figure 4. HIF-1α expression in clinical samples correlates with genes downregulated in HIF-KD sorted tumor cells. A) Correlation plots were drawn using the webtool In Silico Transcriptomics Ver. 2.1.3. (http://ist.medisapiens.com), focusing on 991 human colorectal cancer samples. All results were significant ($p < 0.001$) and positively correlated ($r > 0.2$). **B)** Correlation plots using 594 colorectal cancer patient samples from Colorectal Adenocarcinoma dataset (TCGA, PanCancer Atlas; https://www.cbioportal.org/ ver. 3.4.3).

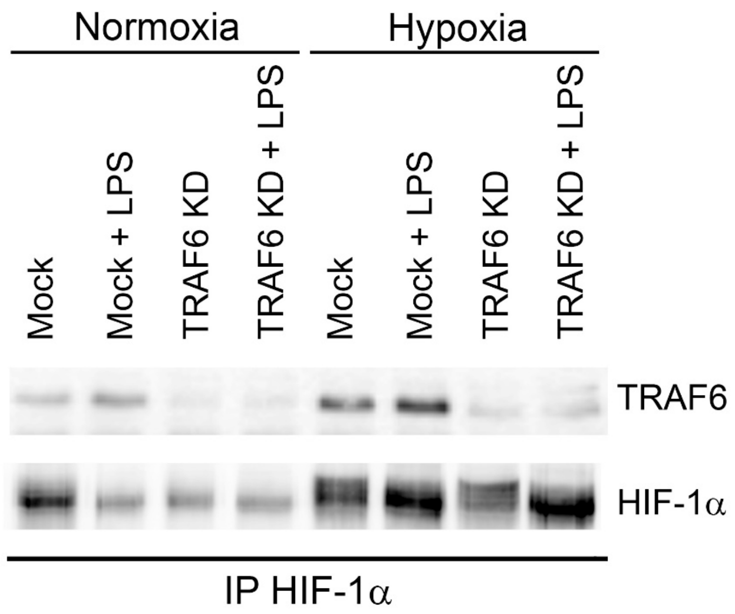

Supplementary Figure 5. HIF-1α immunoprecipitation followed by TRAF-6 detection in murine colon carcinoma cell line MC-38 cells. Immunoprecipitation of HIF-1α in Mock and TRAF6-KD MC-38 cells, which were detected either with TRAF6 or HIF-1α Ab, respectively (n =3). Cells were incubated for 8 hours under normoxia (21% O_2) or hypoxia (0.2% O_2). Additionally, cells were stimulated with 1 μg/mL LPS for one hour either under normoxia or hypoxia.

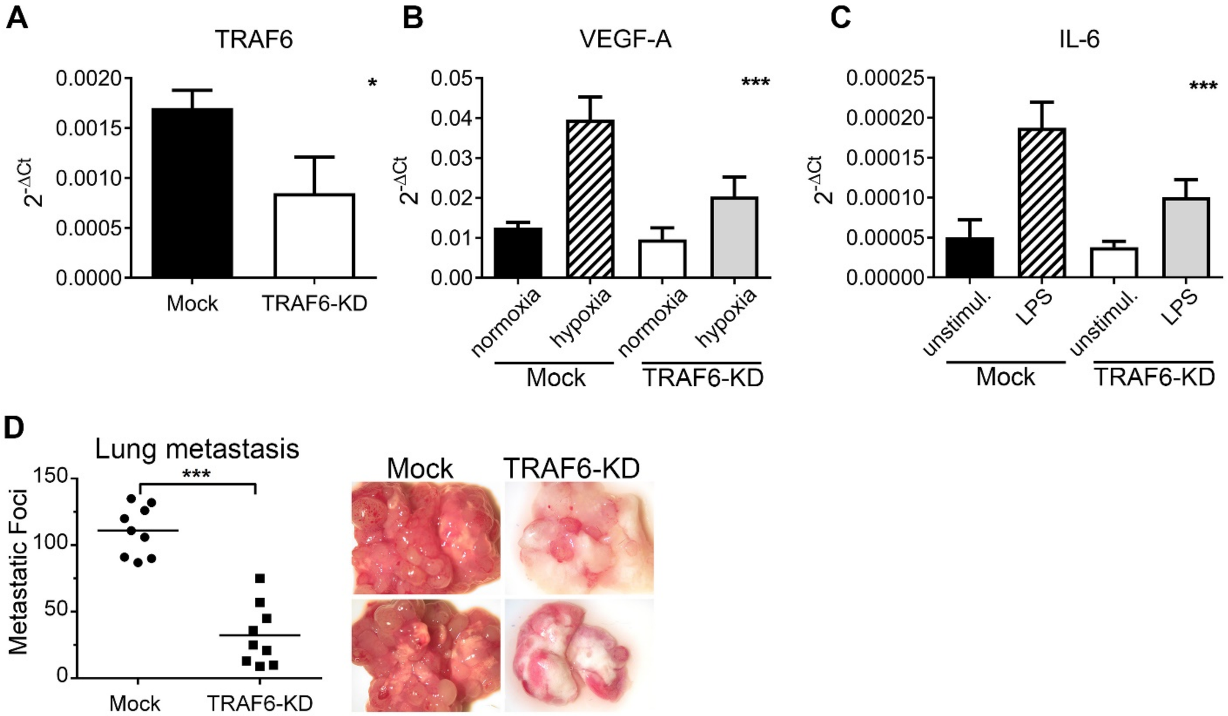

Supplementary Figure 6. Characterization of TRAF6-KD MC-38 cells. A). Transcript levels of TRAF6 were determined by RT-qPCR. mRNA expression levels of VEGF-A **(B)** and canonical TRAF6 target gene IL-6 **(C)** were quantified by RT-qPCR after LPS stimulation (1 μg/mL, 1 hour) compared to mock control and unstimulated cells. Expression levels were normalized to constitutively expressed ribosomal protein S12 mRNA levels. **D)** Experimental lung metastasis of Mock and TRAF6-KD MC-38 cells together with representative images of lung tumors after 28 days of i.v. injection. Mice from two independent experiments are presented. Statistical significance was assessed using the Mann-Whitney test; *, $p < 0.05$; ***, $p < 0.001$.

Supplemental experimental procedures

Angiogenesis Array

Perfused tumors were homogenized in 350 μl PBS containing protease inhibitors (complete EDTA-free, Roche) by using a Polytron (Brinkhan). Samples were spun down (15,000 x g for 15 min at 4°C), supernatant was separated, and protein concentration was determined by BCA assay. Tumor lysate (400 μg) was incubated on a membrane spotted with the Mouse Angiogenesis Antibody Array (R&D Systems). Upon development, pixel density was analyzed with ImageQuant (GE Healthcare Life Sciences). Background signal density was subtracted and normalized to average density of reference spots. Up- and down-regulation of angiogenic factors was analyzed with a R-based web application developed at the Functional Genomics Center Zurich (http://fgcz-shiny.uzh.ch/fgcz_heatmap_app/).

Experimental metastasis

Mice were intravenously (i.v.) injected with 300,000 MC-38GFP Mock or TRAF6-KD cells. Mice were sacrificed after 28 days and lungs were perfused with PBS. The number of metastatic foci per lung was determined macroscopically by a blinded investigator.

RT-qPCR

Total cellular RNA was extracted using TRI Reagent (Sigma) or RNeasy Plus Mini Kit (Qiagen) following manufacturer's recommendations. cDNA was synthesized from 1 μg of total RNA using Omniscript RT Kit (Qiagen). Quantitative qPCR was performed using KAPA SYBR Green Master Mix (Sigma) on a CFX96 Real-Time System (Biorad). Primers were purchased from Microsynth.

Primer	Sequence
HIF-1a forward	5'-CATCCATGTGACCATGAGGA-3'
HIF-1a reverse	5'-CACGTTGCTGACTTGATGTT-3'
VEGF-A forward	5'-GTACCTCCACCATGCCAAGT-3'
VEGF-A reverse	5'-TCTCGTCGGGGTACTCCTGG-3'
CA-IX (Car9) forward	5'-GCCGCTACTACCGATATGAA-3'
CA-IX (Car9) reverse	5'-CACAAGGAAACGGAGAGAGT-3'
TRAF6 forward	5'-TCATCAGAGAACAGATGCCTAAT-3'
TRAF6 reverse	5'-TCATGTGCAACTGGGTATTCT-3'
IL-6 forward	5'-GTGGAAATGAGAAAAGAGTTGTGC-3'
IL-6 reverse	5'-ACCAGAGGAAATTTTCAATAGGC-3'
Cyr61 forward	5'-AGAGGCTTCCTGTCTTTGG-3'
Cyr61 reverse	5'-CACTCTGGGTTGTCATTGGT-3'
Pdgfa forward	5'-GATGAGGACCTGGGCTTGC-3'
Pdgfa reverse	5'-TCAGCCCCTACGGAGTCTATC-3'
Cox2 forward	5'-ACTGGGCCATGGAGTGGA-3'
Cox2 reverse	5'-GAGTGTCTTTGACTGTGGGGG-3'
Tnfrsf9 forward	5'-GCTGCCCTGAGATCGAAA-3'
Tnfrsf9 reverse	5'-GAAAGTACCAGGCTGACAGTTA-3'
S12 forward	5'-ACGTCAACACTGCTCTACAA-3'
S12 reverse	5'-CTCCACCAGCTTGACATACA-3'

Table 1. Differently regulated genes in sorted HIF-KD tumor cells vs. sorted Mock cells.

Upregulated genes

Gene	log2 Ratio	p Value	Gene	log2 Ratio	p Value	Gene	log2 Ratio	p Value
Arsi	1.536	1.95E-08	*Has2*	0.7751	0.004355	*Gm20489*	0.6574	0.006045
Enpp2	1.515	3.13E-09	*Etv1*	0.7745	0.000191	*Il1b*	0.6566	0.01597
Map1a	1.462	3.08E-08	*Tnfrsf21*	0.7729	0.000628	*Cenpe*	0.6563	0.01369
Col18a1	1.343	2.56E-08	*Stard10*	0.7709	0.004949	*Kirrel3*	0.6562	0.0138
Khdrbs3	1.239	3.55E-06	*Ptpn3*	0.7693	0.001993	*Chd5*	0.6556	0.01661
Tmem54	1.234	1.54E-06	*Ptpn6*	0.7674	0.003255	*Efnb1*	0.6549	0.006815
Nol3	1.168	1.57E-05	*Sytl4*	0.7672	0.004688	*Srpx*	0.6547	0.004553
Lrrc15	1.151	2.45E-05	*Cspg4*	0.7628	0.003232	*Mast2*	0.6531	0.002916
Pcsk9	1.11	3.54E-06	*Plxnd1*	0.7598	0.000469	*Zbed4*	0.6531	0.009298
Fas	1.106	4.91E-06	*Rbm19*	0.7595	0.000418	*Camsap1*	0.6519	0.000963
Stmn2	1.104	5.38E-05	*Ednra*	0.759	0.004118	*Akap13*	0.6518	0.003915
Arap3	1.096	1.47E-05	*Bcas1*	0.7585	0.001786	*Ptpn13*	0.6504	0.01112
Slc15a3	1.086	6.18E-06	*Dkk2*	0.7578	0.002947	*Heg1*	0.6492	0.001532
Hs6st1	1.082	8.08E-07	*Gm6880*	0.7522	0.001189	*Sertad4*	0.648	0.002755
Ston2	1.069	4.03E-05	*Pogk*	0.7514	1.39E-05	*Gm684*	0.647	0.01785
Wfdc12	1.063	8.14E-05	*Gdpd5*	0.7507	0.002728	*Spef1*	0.6461	0.01251
Tenm3	1.06	2.04E-05	*Unc5b*	0.7486	0.001513	*S1pr1*	0.6457	0.003973
Card10	1.057	0.000102	*Macf1*	0.7476	0.001448	*Ccr2*	0.6437	0.01544
Duox1	1.022	0.000161	*Apol6*	0.7464	0.006062	*Kctd12*	0.6436	0.01719
Chst2	1.015	2.25E-05	*Tep1*	0.7463	0.004387	*Gfra2*	0.6431	0.005127
Mdn1	1.001	2.02E-05	*Cep290*	0.7453	0.005887	*Dock4*	0.6424	0.003511
Col2a1	0.9928	0.000176	*Rgl1*	0.7432	0.003597	*Nav1*	0.6419	0.002911
Afap1l2	0.9886	5.15E-05	*Kif26b*	0.7411	0.000364	*Abca1*	0.6418	0.001482
Fam198b	0.9873	0.000312	*Dagla*	0.7402	0.001675	*Zfml*	0.641	0.001805
Extl1	0.985	1.16E-05	*Akap9*	0.74	0.004829	*Ssh2*	0.6406	0.002281
Camk2a	0.9764	0.000325	*Gbp5*	0.7381	0.006021	*Stat4*	0.639	0.01567
Arhgap26	0.9761	1.47E-05	*Ryr2*	0.738	0.006528	*Cd276*	0.6384	0.000482
Prodh	0.9698	9.07E-05	*Ncam1*	0.7365	0.004315	*Abi3bp*	0.6369	0.01008
Serpinf1	0.9674	1.08E-07	*Nin*	0.7331	0.00072	*Pcyox1l*	0.6365	0.01231
Slc22a23	0.9619	0.000451	*Lama4*	0.7304	0.006208	*Myh3*	0.6361	0.01906
Nid2	0.9604	0.000284	*Arid5a*	0.73	0.002825	*Fbln5*	0.6351	0.01692
Aldh1a3	0.9577	0.000477	*Adgra3*	0.7294	0.000428	*C1qtnf1*	0.6347	0.01604

Ttn	0.9508	0.000385	*Car14*	0.7294	0.007353	*Slc26a9*	0.6333	0.01604
2900026 A02Rik	0.9474	0.000179	*Neto2*	0.7267	0.001744	*Slitrk5*	0.6314	0.02081
Ptn	0.9462	0.000295	*Zfp600*	0.7254	0.003771	*Fgfr3*	0.63	0.02071
Icam1	0.9447	0.000345	*Tex13*	0.7242	0.00038	*Rgs5*	0.6283	0.02132
Dppa2	0.9439	4.25E-05	*Myo18a*	0.723	0.001961	*Hip1*	0.624	0.003119
Gxylt2	0.9403	0.000157	*Ciita*	0.7177	0.003535	*Zfc3h1*	0.6232	0.004891
Cyp7b1	0.9345	0.000267	*Nckap5*	0.7155	0.009098	*Eml5*	0.6227	0.02289
Cd200	0.9345	0.000439	*Pstpip2*	0.7143	0.008768	*Uhrf1bp1*	0.6222	0.00068
Apcdd1	0.9304	0.000658	*Tcn2*	0.714	0.002885	*Dennd3*	0.6222	0.01726
Scml4	0.9222	0.000547	*Plxnb1*	0.7139	0.002781	*Wisp1*	0.6217	0.001894
Mdfi	0.9219	0.000647	*Cep162*	0.7117	0.00327	*Apobr*	0.6217	0.007581
Ctxn1	0.9142	0.000354	*Nhs*	0.7106	0.009585	*Lpar1*	0.6199	0.01469
Gli1	0.9086	0.000273	*Atp2b4*	0.7098	0.005786	*Golga3*	0.6197	0.001715
Xylt1	0.9067	0.000615	*Nlrc5*	0.7096	0.00061	*Creb3l1*	0.6192	0.01968
Mid1	0.8983	4.39E-05	*Chrnd*	0.7068	0.005292	*Urb1*	0.6184	0.001338
Sorcs2	0.8877	0.000265	*Crip1*	0.7064	0.00975	*Sh3pxd2 a*	0.6182	0.004064
Ank1	0.8854	0.000269	*Xlr4b*	0.7056	0.007636	*Dab2*	0.6179	0.007381
Ceacam 20	0.8782	0.00047	*Prokr1*	0.7042	0.009533	*Col27a1*	0.6173	0.01624
Spib	0.8771	0.001236	*Slfn9*	0.7029	0.001994	*Nfkbie*	0.6165	0.003431
Mov10l1	0.8757	0.001091	*Apc*	0.702	0.000794	*Gfpt2*	0.6162	0.02175
Bace2	0.8751	0.000619	*Slc16a9*	0.7007	0.0106	*Ankrd26*	0.6154	0.005389
Pgbd5	0.8729	0.001324	*Ankrd11*	0.6984	0.006901	*Tmem17 3*	0.615	0.001167
Lama5	0.8723	7.65E-05	*Arap2*	0.6976	0.01055	*Penk*	0.6145	0.0161
Slc4a5	0.872	0.00141	*Tvp23a*	0.6949	0.005427	*Ccdc24*	0.6142	0.00579
Tspan2	0.8641	0.001211	*Fyco1*	0.6929	0.001719	*Gm1315 1*	0.6134	0.02535
Amigo2	0.8617	0.000136	*Lbx1*	0.689	0.00907	*Serpina3 f*	0.6131	0.0152
Crb2	0.8587	0.00113	*2010005 H15Rik*	0.689	0.012	*Myo1e*	0.6129	0.005001
Golgb1	0.8541	0.001176	*Bcl2*	0.6878	0.004286	*Abca13*	0.6127	0.01598
Prune2	0.8463	5.17E-06	*Cd9*	0.6875	0.00227	*Rgma*	0.6122	0.01227
Slamf8	0.843	0.000239	*Rabl6*	0.687	0.00023	*Wnt6*	0.6121	0.02132
Col3a1	0.841	5.03E-05	*Adamtsl3*	0.6864	0.00092	*Tlr12*	0.6115	0.02116
Cd248	0.8396	0.000449	*Dppa4*	0.6861	0.01066	*Baz1b*	0.6106	0.000965
Zbtb40	0.839	0.000141	*Lynx1*	0.6855	0.005872	*Synj2*	0.6095	0.003389
Map3k5	0.8384	0.000552	*Tmem2*	0.6848	0.00245	*Slit2*	0.6092	0.001397
Mlph	0.8372	0.000556	*Il2rg*	0.684	0.004172	*Sema4a*	0.6087	0.01751
Bptf	0.8289	0.000474	*Gm1314 5*	0.6822	0.002066	*Zfp251*	0.608	0.00658

Plxna1	0.8279	0.000332	*Scara5*	0.6821	0.005129	*Dennd2a*	0.6078	0.00335
Vegfc	0.826	0.001686	*Gdf11*	0.6818	0.000524	*Thbs2*	0.6078	0.01377
Notch1	0.8257	0.000457	*Flrt2*	0.6817	0.003201	*P2rx7*	0.6076	0.01264
Mmp28	0.823	0.000812	*Slpi*	0.6802	0.006398	*Tmem159*	0.6076	0.02675
Casp8ap2	0.8171	0.000276	*Mtcl1*	0.6797	0.01184	*Spata13*	0.6048	0.01287
Slx4	0.8147	0.000411	*Itga1*	0.679	0.01299	*Akna*	0.6026	0.01763
Plxna2	0.8112	0.000271	*Pid1*	0.6777	0.006235	*Flrt3*	0.6013	0.02414
Gm15386	0.8108	0.002021	*Tph2*	0.6777	0.01221	*Cntrl*	0.6009	0.02364
Sema6a	0.8078	0.00179	*Gareml*	0.6763	0.01362	*Sema3c*	0.5988	0.02472
Hsph1	0.8065	4.91E-05	*Golga4*	0.6755	0.007958	*Wdfy3*	0.5976	0.004101
Ccdc36	0.8054	0.002731	*Fat4*	0.6754	0.006058	*Grip1*	0.5972	0.0281
Rorb	0.8051	0.002187	*Trak2*	0.6751	0.000293	*Col11a2*	0.5947	0.02764
Abca3	0.805	0.003169	*Col1a1*	0.6743	0.01253	*Slc2a13*	0.5941	0.01877
Pde2a	0.8045	0.002227	*Syne2*	0.674	0.008263	*Uchl1*	0.5938	0.0213
Trpa1	0.7991	0.002311	*Rps6kc1*	0.6714	0.005068	*Trim56*	0.5928	0.007752
Zfp936	0.7985	0.003183	*Chd7*	0.6703	0.0145	*Lck*	0.5928	0.02781
Trip11	0.7963	0.000364	*A4galt*	0.6693	0.01468	*Bdp1*	0.5927	0.007637
Col5a2	0.7926	0.000193	*Nov*	0.6667	0.005249	*Lgi4*	0.5925	0.02681
Cd38	0.7915	0.000715	*Magi1*	0.6663	0.01477	*St3gal1*	0.5923	0.007066
Tmem119	0.7903	0.00275	*Gli2*	0.6633	0.01559	*Tnrc6c*	0.592	0.01445
Spry1	0.79	0.003254	*Nipbl*	0.6615	0.001738	*Serpinh1*	0.5919	0.01216
Amot	0.7884	0.000381	*Tcf20*	0.6602	0.002459	*Ccne1*	0.5915	0.007431
Fjx1	0.7831	0.003726	*Pprc1*	0.6595	0.001264	*C2cd2*	0.5905	0.006618
Lbp	0.7794	0.003456	*Nol4l*	0.6586	0.01597	*Lamc1*	0.5902	0.003146
Pdgfa	0.7786	0.0003	*Ccr7*	0.6578	0.01648	*Fam212b*	0.5901	0.0109
Cntnap4	0.776	0.004216						

Downregulated genes

**Gene**	**log2 Ratio**	**p Value**	**Gene**	**log2 Ratio**	**p Value**	**Gene**	**log2 Ratio**	**p Value**
Tmem218	-0.5912	0.008697	*Sorbs3*	-0.6919	0.000315	*Pgam1*	-0.9223	1.69E-09
Nusap1	-0.5912	0.01872	*Ctla2a*	-0.6923	0.004933	*Plac8*	-0.9269	8.39E-06
Rpl36	-0.5924	0.009233	*Gpr146*	-0.6931	0.002179	*Wwc1*	-0.9277	0.00068
Deptor	-0.5941	0.02758	*Hbegf*	-0.6945	0.004465	*Cdh17*	-0.9292	0.000614
Cd109	-0.5947	0.01985	*Amotl2*	-0.6951	0.005257	*Pttg1ip*	-0.9315	1.50E-06
Nmrk1	-0.5949	0.006166	*Galnt13*	-0.6961	0.01007	*Bhlhe40*	-0.932	5.69E-06
Cdkn2d	-0.5956	0.00502	*S100a3*	-0.6971	0.009731	*Hist1h1c*	-0.9428	0.000132
Tinagl1	-0.5958	0.02542	*Sgcd*	-0.6979	0.01064	*Cdkn1b*	-0.9468	1.34E-06
Alkbh5	-0.596	0.00065	*Upk1b*	-0.6985	0.00771	*Junb*	-0.9502	0.00017
Akap7	-0.596	0.007963	*Pqlc3*	-0.6997	0.002683	*Mfsd9*	-0.9629	0.000182
Cyp4v3	-0.597	0.009686	*Galk1*	-0.703	0.000106	*3110057O12Rik*	-0.9651	0.00021
Ppp1r3b	-0.5979	0.02211	*Map2k1*	-0.7097	6.11E-05	*Foxc2*	-0.9682	0.000248
Klf10	-0.5983	0.005653	*Timp3*	-0.7115	0.007006	*Fabp5*	-0.9747	2.12E-05
Eps8l2	-0.5988	0.001111	*Usp53*	-0.7147	0.002861	*Klf6*	-0.9787	1.06E-05
Hpse	-0.5991	0.001167	*Msln*	-0.7186	0.006922	*Ampd3*	-0.9804	9.13E-07
Pxmp4	-0.6003	0.008968	*Srd5a2*	-0.719	0.007495	*Tpi1*	-0.9825	1.33E-10
Slc37a4	-0.6015	0.001067	*G2e3*	-0.7195	0.001169	*Gpi1*	-0.9833	1.12E-08
B630005N14Rik	-0.6016	0.003596	*Als2cr12*	-0.7218	0.008446	*Gapdh*	-1.003	4.01E-10
Ccdc109b	-0.6043	0.002009	*Hif1a*	-0.7225	0.008222	*U90926*	-1.014	0.000214
Sat1	-0.6044	0.002473	*Neurl2*	-0.7227	0.004352	*Sertad1*	-1.018	1.33E-07
Lxn	-0.6044	0.01426	*Pdk3*	-0.725	6.28E-05	*Serpine1*	-1.018	2.55E-06
Adm	-0.6054	0.02169	*Tcp11l2*	-0.7253	0.007003	*Nos2*	-1.02	9.96E-05
Rps10	-0.6055	0.001354	*Dyrk4*	-0.7254	0.008133	*Maff*	-1.023	1.50E-06

Cdc42ep2	-0.6075	0.000723	*Cda*	-0.7294	0.000239	*Egr2*	-1.024	6.83E-05
Rapsn	-0.6083	0.02147	*Rpl22l1*	-0.731	0.001717	*Mctp2*	-1.027	0.00016
Id3	-0.6083	0.02287	*Gria4*	-0.7364	0.001079	*Prdm1*	-1.035	0.000148
Hbp1	-0.6086	0.003932	*Pfkp*	-0.7375	2.31E-05	*Klk1b27*	-1.036	3.95E-05
Islr2	-0.6092	0.02606	*Slc2a8*	-0.7389	0.000931	*Acsbg1*	-1.041	8.02E-05
Rps24	-0.611	0.002705	*Pcgf5*	-0.74	4.11E-05	*Ldha*	-1.043	2.83E-10
Arrdc3	-0.6111	0.001487	*Slc16a6*	-0.74	0.003221	*Hist1h2bc*	-1.047	5.91E-06
Ftl1	-0.612	0.000445	*Txnip*	-0.7472	0.000728	*Aldoc*	-1.048	0.000127
Dnajc5	-0.6123	0.000367	*Klra4*	-0.7472	0.00223	*Ramp3*	-1.051	0.000127
AC136513.6	-0.6126	0.001234	*Plekha2*	-0.7487	6.29E-05	*Epha7*	-1.055	0.000115
Tpt1	-0.6157	0.00177	*Klhl24*	-0.7532	0.000956	*Aire*	-1.057	7.96E-05
Rassf7	-0.6157	0.007976	*Rundc3b*	-0.7536	0.002259	*Ephx1*	-1.058	3.58E-06
Vim	-0.6195	4.68E-05	*Casp12*	-0.7573	0.00143	*Slc16a3*	-1.059	6.22E-09
Eid1	-0.6208	0.002794	*Pkm*	-0.7628	3.64E-07	*Fgf7*	-1.065	1.42E-05
Ube2e2	-0.6219	0.003813	*Socs3*	-0.7643	0.000455	*Zfp36*	-1.069	2.74E-05
Klf9	-0.6236	0.008532	*Hspb6*	-0.7665	0.000453	*Aldoa*	-1.083	3.13E-10
Pik3ip1	-0.6249	0.004236	*Eno1*	-0.7693	3.88E-06	*Hacd2*	-1.093	3.43E-07
Rusc2	-0.6252	0.009511	*Pik3cb*	-0.7709	0.000114	*Lox*	-1.094	4.97E-05
Homer1	-0.6266	0.002161	*Nampt*	-0.7743	4.03E-05	*Klf11*	-1.102	1.12E-06
Insig2	-0.628	0.001689	*Fam46b*	-0.7752	0.00352	*Pfkl*	-1.108	1.43E-09
Nicn1	-0.6281	0.002307	*Mturn*	-0.7782	0.004539	*Car9*	-1.109	4.54E-05
Cryl1	-0.6339	0.01577	*Nxph4*	-0.782	0.002749	*Ier2*	-1.114	6.95E-07
Trim17	-0.634	0.007392	*Hist3h2a*	-0.7823	0.000559	*Frat2*	-1.137	2.11E-05
Hk1	-0.6345	5.64E-05	*Ndufv3*	-0.7826	6.38E-07	*Lrrn4cl*	-1.138	1.08E-06
Cnr1	-0.635	0.007941	*Lgals3*	-0.7834	0.00076	*Higd1a*	-1.164	5.21E-09

Myl9	-0.6363	0.01413	*Tiparp*	-0.7899	0.00028	*Pgm2*	-1.168	2.82E-11
Peli1	-0.6371	0.0107	*Pnrc1*	-0.7907	0.000618	*Mxi1*	-1.171	1.47E-10
Cyp2d22	-0.6375	0.004964	*Dmxl1*	-0.7911	0.000796	*Crebrf*	-1.175	2.39E-06
Eef2	-0.638	0.00011	*Hoga1*	-0.7927	0.001347	*Rhob*	-1.185	6.83E-08
Ugdh	-0.6381	0.000254	*Il13ra2*	-0.7933	0.003739	*Hlf*	-1.209	9.88E-06
Inpp5k	-0.6393	0.003238	*Ddit4*	-0.7973	8.55E-06	*Mgarp*	-1.211	2.93E-09
Gsto2	-0.6397	0.01707	*Sgk1*	-0.801	4.59E-05	*Jund*	-1.214	7.84E-08
Dffb	-0.6406	0.008095	*Tmem171*	-0.803	0.003157	*Sap30*	-1.219	6.04E-10
Cdh5	-0.6411	0.004012	*Mif*	-0.8049	6.88E-05	*Itpk1*	-1.235	4.57E-11
Me2	-0.643	0.000158	*Elovl7*	-0.8071	0.000132	*Celf2*	-1.241	2.31E-08
Nsun3	-0.6436	0.00725	*Jun*	-0.8105	0.000443	*Ak4*	-1.256	1.62E-11
Otud1	-0.6439	0.01015	*Lrrn3*	-0.812	0.002258	*Klf2*	-1.261	1.78E-07
Itgb7	-0.6442	0.006606	*Fhl2*	-0.8138	0.000196	*Ciart*	-1.273	5.47E-08
Spg21	-0.6447	0.000738	*Cyr61*	-0.817	0.000687	*Gys1*	-1.279	3.36E-14
Lactb2	-0.6447	0.001587	*Ptgs2*	-0.825	3.91E-05	*Prelid2*	-1.299	6.46E-10
Agl	-0.6488	0.001925	*Cirbp*	-0.8266	0.000188	*Angptl4*	-1.326	6.60E-07
Gpr137b	-0.6504	0.002221	*Medag*	-0.8282	0.000417	*Ppp1r3c*	-1.326	1.09E-06
Prkab2	-0.651	0.000225	*1500012F01Rik*	-0.8327	0.000797	*Pdk1*	-1.328	4.88E-11
Pim1	-0.6511	0.005347	*Sdc4*	-0.8346	5.88E-07	*Epm2a*	-1.332	7.19E-07
Rnf217	-0.6521	0.000996	*Dusp10*	-0.8355	0.000648	*Gbe1*	-1.335	4.73E-08
Fosl1	-0.6555	0.002323	*Wisp2*	-0.8357	0.002311	*Fam162a*	-1.339	3.13E-12
Pde4d	-0.6574	0.003266	*Egln3*	-0.8368	0.00219	*Klk8*	-1.339	3.10E-07
Ankrd1	-0.6581	0.006869	*Kdm4b*	-0.8376	2.49E-05	*Tnfrsf9*	-1.354	1.14E-07
Acp6	-0.6599	0.000703	*Rbm3*	-0.8406	2.16E-05	*Dhrs3*	-1.356	2.19E-08
Pax9	-0.6611	0.007406	*Dusp4*	-0.8412	0.001316	*Il33*	-1.371	2.98E-08

Zc3h6	-0.664	0.01232	*Adam8*	-0.8434	3.58E-05	*Pgk1*	-1.373	1.29E-12
Chil3	-0.6642	0.009666	*Srgap3*	-0.8478	0.001996	*Ndrg1*	-1.379	1.82E-12
Plod2	-0.665	0.000192	*Inpp4b*	-0.8496	9.85E-05	*Cdkn1a*	-1.386	7.33E-12
Figf	-0.669	0.005873	*Btg2*	-0.8506	0.001491	*Mt2*	-1.387	4.26E-11
Crlf1	-0.6699	0.009754	*Herc3*	-0.8566	0.001592	*Per1*	-1.398	5.28E-10
Fam117b	-0.6727	0.000182	*2610528A11Rik*	-0.8567	0.001303	*Bnip3l*	-1.412	8.59E-12
Myo1h	-0.6729	0.01367	*Xdh*	-0.8626	0.000276	*Ccng2*	-1.46	4.84E-11
Foxg1	-0.6741	0.004553	*Rap2c*	-0.8653	0.000167	*Egln1*	-1.464	1.37E-16
Bsg	-0.6756	7.07E-05	*Zfp395*	-0.8679	0.000391	*Mt1*	-1.489	7.35E-16
Acap1	-0.6769	0.01348	*Itga10*	-0.8683	0.00136	*F3*	-1.492	1.65E-11
Appl2	-0.6775	0.001094	*Rcor2*	-0.8685	1.01E-06	*Prl2c2*	-1.504	2.66E-09
Csrnp1	-0.6779	0.002688	*Ctnnal1*	-0.8729	8.88E-05	*Hilpda*	-1.516	2.39E-13
Cav2	-0.6783	0.001056	*Abcg2*	-0.877	0.000658	*Slc2a1*	-1.543	4.68E-13
Gng2	-0.6783	0.009564	*Sema3f*	-0.8781	6.16E-05	*Selenbp1*	-1.567	3.43E-12
Lypd8	-0.6789	0.01081	*Dusp1*	-0.8789	0.001081	*Hmox1*	-1.6	2.72E-12
Trib1	-0.682	0.002135	*Fosl2*	-0.8864	1.29E-07	*Mpp2*	-1.601	2.32E-10
Pafah1b3	-0.6831	6.93E-05	*Trappc6a*	-0.8884	7.16E-05	*Ier3*	-1.644	1.07E-14
Btg1	-0.6847	0.000143	*Gm11110*	-0.8939	0.000429	*Ero1l*	-1.677	2.43E-15
Sdpr	-0.6855	0.003124	*Gpr35*	-0.8982	0.000933	*Ndufa4l2*	-1.813	2.81E-16
Adam22	-0.6862	0.01062	*Haghl*	-0.9003	0.000282	*Apln*	-1.841	4.22E-16
Serpinb9g	-0.6899	0.004293	*Dusp5*	-0.9142	0.000107	*Ankrd37*	-1.881	4.00E-15
Bnc1	-0.6903	0.007763	*Grhpr*	-0.9148	5.56E-08	*Nrn1*	-2.209	4.20E-18
Hoxb13	-0.6909	0.00556	*Pi15*	-0.9182	0.000224	*Bnip3*	-2.211	9.17E-25

Supplementary Figure 7. Predicted target genes for hsa-miR-29a-3p, overlapping with up- and down-regulated genes identified in sorted MC-38 cells in sorted HIF-KD tumor cells vs. sorted Mock cells. hsa-miR-29a-3p [1] target genes were identified using MicroRNA Data Integration Portal, mirDIP version 4.1.11.1 (Database version 4.1.0.3) (http://ophid.utoronto.ca/mirDIP) [2], using all 30 datasets, high confidence level, and bi-directional search option. While the table lists all known sources for individual genes, there are 81 unique up-regulated targets and 87 unique down-regulated targets.

Upregulated Gene targeted by mir29

	Gene Symbol	Uniprot	Source	Confidence class
UP	**ABCA13**	Q86UQ4	microrna.org	High
UP	**ADGRA3**	Q8IWK6	microrna.org	Very High
UP	**AKAP13**	Q12802	CoMeTa	High
UP	**AKAP13**	Q12802	ElMMo3	Very High
UP	**AKAP13**	Q12802	MultiMiTar	High
UP	**AKAP13**	Q12802	PACCMIT	High
UP	**AKAP13**	Q12802	TargetRank	Very High
UP	**AKAP13**	Q12802	TargetScan	Very High
UP	**AKAP13**	Q12802	miRTar2GO	High
UP	**AKAP13**	Q12802	MirAncesTar	High
UP	**AKAP13**	Q12802	microrna.org	High
UP	**AMOT**	Q4VCS5	CoMeTa	High
UP	**AMOT**	Q4VCS5	Cupid	High
UP	**AMOT**	Q4VCS5	ElMMo3	Very High
UP	**AMOT**	Q4VCS5	MAMI	High
UP	**AMOT**	Q4VCS5	MultiMiTar	High
UP	**AMOT**	Q4VCS5	PACCMIT	High
UP	**AMOT**	Q4VCS5	TargetRank	Very High
UP	**AMOT**	Q4VCS5	TargetScan	Very High
UP	**AMOT**	Q4VCS5	miRTar2GO	High
UP	**AMOT**	Q4VCS5	microrna.org	High
UP	**ANK1**	P16157	CoMeTa	High
UP	**ANK1**	P16157	Cupid	Very High
UP	**ANK1**	P16157	ElMMo3	Very High
UP	**ANK1**	P16157	PACCMIT	High
UP	**ANK1**	P16157	TargetScan	Very High
UP	**APC**	P25054	TargetScan	Very High
UP	**APC**	P25054	microrna.org	Very High
UP	**APOL6**	Q9BWW8	miRTar2GO	High
UP	**ARAP2**	Q8WZ64	microrna.org	High

UP	ARAP3	Q8WWN8	CoMeTa	High
UP	ARAP3	Q8WWN8	ElMMo3	High
UP	ARAP3	Q8WWN8	MAMI	High
UP	ARAP3	Q8WWN8	microrna.org	High
UP	ATP2B4	P23634	CoMeTa	Very High
UP	ATP2B4	P23634	Cupid	Very High
UP	ATP2B4	P23634	ElMMo3	Very High
UP	ATP2B4	P23634	MultiMiTar	High
UP	ATP2B4	P23634	PACCMIT	High
UP	ATP2B4	P23634	TargetRank	High
UP	ATP2B4	P23634	TargetScan	Very High
UP	ATP2B4	P23634	miRTar2GO	High
UP	ATP2B4	P23634	microrna.org	High
UP	BCL2	P10415	CoMeTa	High
UP	BDP1	A6H8Y1	miRTar2GO	High
UP	BPTF	Q12830	Cupid	Very High
UP	BPTF	Q12830	ElMMo3	High
UP	CAMK2A	Q9UQM7	MultiMiTar	High
UP	CAMSAP1	Q5T5Y3	miRTar2GO	High
UP	CCDC36	Q8IYA8	MAMI	High
UP	CCDC36	Q8IYA8	microrna.org	Very High
UP	CCNE1	P24864	Cupid	Very High
UP	CCNE1	P24864	ElMMo3	High
UP	CCNE1	P24864	miRTar2GO	High
UP	CD276	Q5ZPR3	ElMMo3	Very High
UP	CD276	Q5ZPR3	MAMI	High
UP	CD276	Q5ZPR3	PACCMIT	High
UP	CD276	Q5ZPR3	TargetRank	Very High
UP	CD276	Q5ZPR3	TargetScan	Very High
UP	CD276	Q5ZPR3	miRTar2GO	High
UP	CD276	Q5ZPR3	microrna.org	High
UP	COL1A1	P02452	CoMeTa	High
UP	COL1A1	P02452	Cupid	Very High
UP	COL1A1	P02452	DIANA	High
UP	COL1A1	P02452	ElMMo3	Very High
UP	COL1A1	P02452	GenMir++	Very High
UP	COL1A1	P02452	MAMI	Very High
UP	COL1A1	P02452	Mirza-G	High
UP	COL1A1	P02452	MultiMiTar	High
UP	COL1A1	P02452	PACCMIT	High
UP	COL1A1	P02452	TargetRank	High
UP	COL1A1	P02452	TargetScan	Very High
UP	COL1A1	P02452	miRTar2GO	High
UP	COL1A1	P02452	microrna.org	High
UP	COL27A1	Q8IZC6	DIANA	High
UP	COL27A1	Q8IZC6	PACCMIT	High

UP	**COL27A1**	Q8IZC6	TargetScan	Very High
UP	**COL2A1**	P02458	CoMeTa	High
UP	**COL2A1**	P02458	DIANA	Very High
UP	**COL2A1**	P02458	ElMMo3	Very High
UP	**COL2A1**	P02458	MirTar	High
UP	**COL2A1**	P02458	PicTar	Very High
UP	**COL2A1**	P02458	TargetRank	Very High
UP	**COL2A1**	P02458	TargetScan	Very High
UP	**COL2A1**	P02458	miRDB	High
UP	**COL2A1**	P02458	microrna.org	Very High
UP	**COL3A1**	P02461	CoMeTa	Very High
UP	**COL3A1**	P02461	Cupid	Very High
UP	**COL3A1**	P02461	DIANA	Very High
UP	**COL3A1**	P02461	ElMMo3	Very High
UP	**COL3A1**	P02461	MAMI	Very High
UP	**COL3A1**	P02461	MirTar	Very High
UP	**COL3A1**	P02461	Mirza-G	High
UP	**COL3A1**	P02461	MultiMiTar	High
UP	**COL3A1**	P02461	PACCMIT	High
UP	**COL3A1**	P02461	PicTar	Very High
UP	**COL3A1**	P02461	TargetRank	Very High
UP	**COL3A1**	P02461	TargetScan	Very High
UP	**COL3A1**	P02461	miRDB	High
UP	**COL3A1**	P02461	microrna.org	Very High
UP	**COL5A2**	P05997	CoMeTa	Very High
UP	**COL5A2**	P05997	Cupid	Very High
UP	**COL5A2**	P05997	DIANA	High
UP	**COL5A2**	P05997	ElMMo3	Very High
UP	**COL5A2**	P05997	MirTar	High
UP	**COL5A2**	P05997	Mirza-G	High
UP	**COL5A2**	P05997	PACCMIT	High
UP	**COL5A2**	P05997	PicTar	Very High
UP	**COL5A2**	P05997	TargetScan	Very High
UP	**COL5A2**	P05997	miRDB	High
UP	**COL5A2**	P05997	miRTar2GO	High
UP	**COL5A2**	P05997	microrna.org	Very High
UP	**CSPG4**	Q6UVK1	ElMMo3	High
UP	**CSPG4**	Q6UVK1	MAMI	High
UP	**CSPG4**	Q6UVK1	PACCMIT	High
UP	**CSPG4**	Q6UVK1	TargetScan	Very High
UP	**CSPG4**	Q6UVK1	microrna.org	High
UP	**DENND3**	A2RUS2	microrna.org	High
UP	**EML5**	Q05BV3	Cupid	Very High
UP	**EML5**	Q05BV3	DIANA	High
UP	**EML5**	Q05BV3	ElMMo3	Very High
UP	**EML5**	Q05BV3	MAMI	High

UP	**EML5**	Q05BV3	PACCMIT	High
UP	**EML5**	Q05BV3	TargetScan	Very High
UP	**EML5**	Q05BV3	miRDB	High
UP	**EML5**	Q05BV3	miRTar2GO	High
UP	**EML5**	Q05BV3	microrna.org	High
UP	**ENPP2**	Q13822	CoMeTa	High
UP	**ENPP2**	Q13822	Cupid	Very High
UP	**ENPP2**	Q13822	TargetRank	Very High
UP	**ENPP2**	Q13822	TargetScan	Very High
UP	**ENPP2**	Q13822	microrna.org	Very High
UP	**FAS**	P25445	CoMeTa	High
UP	**FAS**	P25445	microrna.org	High
UP	**GLI2**	P10070	miRTar2GO	High
UP	**GLI2**	P10070	microrna.org	High
UP	**GOLGA3**	Q08378	miRTar2GO	High
UP	**GRIP1**	Q9Y3R0	DIANA	Very High
UP	**GRIP1**	Q9Y3R0	ElMMo3	Very High
UP	**GRIP1**	Q9Y3R0	MAMI	High
UP	**GRIP1**	Q9Y3R0	TargetScan	Very High
UP	**GRIP1**	Q9Y3R0	miRTar2GO	High
UP	**GRIP1**	Q9Y3R0	microrna.org	Very High
UP	**GXYLT2**	A0PJZ3	Cupid	Very High
UP	**GXYLT2**	A0PJZ3	DIANA	High
UP	**GXYLT2**	A0PJZ3	ElMMo3	Very High
UP	**GXYLT2**	A0PJZ3	TargetScan	Very High
UP	**GXYLT2**	A0PJZ3	miRTar2GO	High
UP	**GXYLT2**	A0PJZ3	microrna.org	Very High
UP	**HAS2**	Q92819	TargetScan	Very High
UP	**HIP1**	O00291	CoMeTa	High
UP	**HIP1**	O00291	ElMMo3	High
UP	**HIP1**	O00291	TargetScan	Very High
UP	**HIP1**	O00291	MirAncesTar	High
UP	**KIF26B**	Q2KJY2	CoMeTa	High
UP	**KIF26B**	Q2KJY2	Cupid	Very High
UP	**KIF26B**	Q2KJY2	DIANA	Very High
UP	**KIF26B**	Q2KJY2	ElMMo3	Very High
UP	**KIF26B**	Q2KJY2	MAMI	High
UP	**KIF26B**	Q2KJY2	PACCMIT	High
UP	**KIF26B**	Q2KJY2	TargetRank	Very High
UP	**KIF26B**	Q2KJY2	TargetScan	Very High
UP	**KIF26B**	Q2KJY2	miRDB	High
UP	**KIF26B**	Q2KJY2	microrna.org	Very High
UP	**LAMC1**	P11047	CoMeTa	Very High
UP	**LAMC1**	P11047	DIANA	High
UP	**LAMC1**	P11047	ElMMo3	Very High
UP	**LAMC1**	P11047	MAMI	Very High

UP	LAMC1	P11047	Mirza-G	High
UP	LAMC1	P11047	PACCMIT	High
UP	LAMC1	P11047	TargetRank	Very High
UP	LAMC1	P11047	TargetScan	Very High
UP	LAMC1	P11047	miRDB	High
UP	LAMC1	P11047	MirAncesTar	High
UP	LAMC1	P11047	microrna.org	Very High
UP	LBP	P18428	CoMeTa	High
UP	MAGI1	Q96QZ7	CoMeTa	High
UP	MDN1	Q9NU22	miRTar2GO	High
UP	MMP28	Q9H239	microrna.org	High
UP	MYO18A	Q92614	ElMMo3	High
UP	MYO18A	Q92614	MultiMiTar	High
UP	MYO18A	Q92614	PACCMIT	High
UP	MYO18A	Q92614	TargetScan	Very High
UP	NAV1	Q8NEY1	CoMeTa	High
UP	NAV1	Q8NEY1	Cupid	Very High
UP	NAV1	Q8NEY1	DIANA	High
UP	NAV1	Q8NEY1	ElMMo3	Very High
UP	NAV1	Q8NEY1	MirTar	High
UP	NAV1	Q8NEY1	Mirza-G	High
UP	NAV1	Q8NEY1	MultiMiTar	High
UP	NAV1	Q8NEY1	PACCMIT	High
UP	NAV1	Q8NEY1	TargetRank	High
UP	NAV1	Q8NEY1	TargetScan	Very High
UP	NAV1	Q8NEY1	miRTar2GO	High
UP	NAV1	Q8NEY1	microrna.org	High
UP	NCAM1	P13591	CoMeTa	High
UP	NCAM1	P13591	microrna.org	High
UP	NCKAP5	O14513	Cupid	Very High
UP	NCKAP5	O14513	ElMMo3	Very High
UP	NCKAP5	O14513	MAMI	High
UP	NCKAP5	O14513	Mirza-G	High
UP	NCKAP5	O14513	PACCMIT	High
UP	NCKAP5	O14513	TargetRank	Very High
UP	NCKAP5	O14513	TargetScan	Very High
UP	NCKAP5	O14513	miRDB	High
UP	NCKAP5	O14513	microrna.org	Very High
UP	NID2	Q14112	CoMeTa	High
UP	NID2	Q14112	Cupid	Very High
UP	NID2	Q14112	ElMMo3	Very High
UP	NID2	Q14112	MAMI	High
UP	NID2	Q14112	microrna.org	High
UP	NIN	Q8N4C6	miRTar2GO	High
UP	NIPBL	Q6KC79	microrna.org	High
UP	NOL4L	Q96MY1	Cupid	Very High

UP	NOL4L	Q96MY1	ElMMo3	Very High
UP	NOL4L	Q96MY1	Mirza-G	High
UP	NOL4L	Q96MY1	TargetScan	Very High
UP	NOL4L	Q96MY1	miRTar2GO	High
UP	NOL4L	Q96MY1	MirAncesTar	High
UP	NOL4L	Q96MY1	microrna.org	High
UP	PCSK9	Q8NBP7	miRTar2GO	High
UP	PDGFA	P04085	MultiMiTar	High
UP	PENK	P01210	MAMI	High
UP	PLXNA1	Q9UIW2	CoMeTa	High
UP	PLXNA1	Q9UIW2	ElMMo3	High
UP	PLXNA1	Q9UIW2	MultiMiTar	High
UP	PLXNA1	Q9UIW2	PicTar	Very High
UP	PLXNA1	Q9UIW2	TargetRank	Very High
UP	PLXNA1	Q9UIW2	TargetScan	Very High
UP	PLXNA1	Q9UIW2	miRDB	High
UP	PLXNA1	Q9UIW2	miRTar2GO	High
UP	PLXNA1	Q9UIW2	microrna.org	High
UP	RABL6	Q3YEC7	miRTar2GO	High
UP	RABL6	Q3YEC7	microrna.org	High
UP	SCML4	Q8N228	microrna.org	High
UP	SEMA3C	Q99985	miRTar2GO	High
UP	SEMA6A	Q9H2E6	microrna.org	High
UP	SERPINH1	P50454	CoMeTa	Very High
UP	SERPINH1	P50454	Cupid	Very High
UP	SERPINH1	P50454	DIANA	High
UP	SERPINH1	P50454	ElMMo3	Very High
UP	SERPINH1	P50454	MultiMiTar	High
UP	SERPINH1	P50454	TargetScan	Very High
UP	SERTAD4	Q9NUC0	MirTar	High
UP	SERTAD4	Q9NUC0	TargetRank	High
UP	SERTAD4	Q9NUC0	microrna.org	High
UP	SH3PXD2A	Q5TCZ1	CoMeTa	High
UP	SH3PXD2A	Q5TCZ1	Cupid	Very High
UP	SH3PXD2A	Q5TCZ1	DIANA	Very High
UP	SH3PXD2A	Q5TCZ1	ElMMo3	Very High
UP	SH3PXD2A	Q5TCZ1	Mirza-G	High
UP	SH3PXD2A	Q5TCZ1	PACCMIT	Very High
UP	SH3PXD2A	Q5TCZ1	TargetRank	High
UP	SH3PXD2A	Q5TCZ1	TargetScan	Very High
UP	SH3PXD2A	Q5TCZ1	miRTar2GO	High
UP	SLC2A13	Q96QE2	MultiMiTar	High
UP	SLITRK5	O94991	Cupid	Very High
UP	SPRY1	O43609	CoMeTa	High
UP	SPRY1	O43609	Cupid	Very High
UP	SPRY1	O43609	ElMMo3	Very High

UP	**SPRY1**	O43609	Mirza-G	High
UP	**SPRY1**	O43609	PACCMIT	High
UP	**SPRY1**	O43609	TargetScan	Very High
UP	**SPRY1**	O43609	microrna.org	Very High
UP	**SSH2**	Q76I76	ElMMo3	High
UP	**SSH2**	Q76I76	MultiMiTar	High
UP	**SSH2**	Q76I76	PACCMIT	High
UP	**SSH2**	Q76I76	miRTar2GO	High
UP	**STMN2**	Q93045	DIANA	Very High
UP	**STMN2**	Q93045	ElMMo3	Very High
UP	**STMN2**	Q93045	PACCMIT	High
UP	**STMN2**	Q93045	TargetScan	Very High
UP	**SYNE2**	Q8WXH0	CoMeTa	High
UP	**SYNE2**	Q8WXH0	microrna.org	High
UP	**SYNJ2**	O15056	microrna.org	High
UP	**TENM3**	Q9P273	CoMeTa	High
UP	**TENM3**	Q9P273	Cupid	High
UP	**TENM3**	Q9P273	ElMMo3	Very High
UP	**TENM3**	Q9P273	TargetScan	Very High
UP	**TENM3**	Q9P273	microrna.org	High
UP	**THBS2**	P35442	TargetRank	Very High
UP	**THBS2**	P35442	microrna.org	Very High
UP	**TMEM159**	Q96B96	microrna.org	High
UP	**TNRC6C**	Q9HCJ0	microrna.org	High
UP	**TRAK2**	O60296	ElMMo3	High
UP	**TRAK2**	O60296	MultiMiTar	High
UP	**TRAK2**	O60296	PACCMIT	High
UP	**TRAK2**	O60296	TargetScan	Very High
UP	**TRAK2**	O60296	miRTar2GO	High
UP	**TRAK2**	O60296	microrna.org	High
UP	**TRIM56**	Q9BRZ2	microrna.org	High
UP	**UHRF1BP1**	Q6BDS2	ElMMo3	High
UP	**UHRF1BP1**	Q6BDS2	MultiMiTar	High
UP	**UHRF1BP1**	Q6BDS2	miRTar2GO	High
UP	**UHRF1BP1**	Q6BDS2	microrna.org	High
UP	**UNC5B**	Q8IZJ1	microrna.org	High
UP	**URB1**	O60287	TargetScan	Very High
UP	**WDFY3**	Q8IZQ1	CoMeTa	Very High
UP	**WDFY3**	Q8IZQ1	Cupid	Very High
UP	**WDFY3**	Q8IZQ1	ElMMo3	Very High
UP	**WDFY3**	Q8IZQ1	MultiMiTar	High
UP	**WDFY3**	Q8IZQ1	PACCMIT	High
UP	**WDFY3**	Q8IZQ1	TargetScan	Very High
UP	**WDFY3**	Q8IZQ1	miRTar2GO	High
UP	**WISP1**	O95388	TargetScan	Very High
UP	**WISP1**	O95388	MirAncesTar	High

UP	WISP1	O95388	microrna.org	High
UP	XYLT1	Q86Y38	MultiMiTar	High
UP	ZBTB40	Q9NUA8	CoMeTa	High
UP	ZBTB40	Q9NUA8	Cupid	Very High
UP	ZBTB40	Q9NUA8	ElMMo3	Very High
UP	ZBTB40	Q9NUA8	MAMI	High
UP	ZBTB40	Q9NUA8	MultiMiTar	High
UP	ZBTB40	Q9NUA8	PACCMIT	High
UP	ZBTB40	Q9NUA8	TargetScan	Very High
UP	ZBTB40	Q9NUA8	MirAncesTar	High
UP	ZBTB40	Q9NUA8	microrna.org	High
UP	ZNF251	Q9BRH9	microrna.org	High
UP	ZNF616	Q08AN1	microrna.org	High

Downregulated Gene targeted by mir29

	Gene Symbol	Uniprot	Source	Confidence class
DOWN	ABHD18	Q0P651	TargetScan	Very High
DOWN	ADAM22	Q9P0K1	ElMMo3	Very High
DOWN	ADAM8	P78325	microrna.org	High
DOWN	AK4	P27144	CoMeTa	High
DOWN	AK4	P27144	ElMMo3	High
DOWN	AK4	P27144	microrna.org	High
DOWN	AMPD3	Q01432	MultiMiTar	High
DOWN	AMPD3	Q01432	microrna.org	High
DOWN	ANGPTL4	Q9BY76	CoMeTa	High
DOWN	ANGPTL4	Q9BY76	ElMMo3	High
DOWN	ANGPTL4	Q9BY76	MAMI	Very High
DOWN	ANGPTL4	Q9BY76	microrna.org	High
DOWN	ARRDC3	Q96B67	Cupid	Very High
DOWN	ARRDC3	Q96B67	DIANA	High
DOWN	ARRDC3	Q96B67	ElMMo3	Very High
DOWN	ARRDC3	Q96B67	MirTar	High
DOWN	ARRDC3	Q96B67	Mirza-G	High
DOWN	ARRDC3	Q96B67	MultiMiTar	High
DOWN	ARRDC3	Q96B67	PACCMIT	High
DOWN	ARRDC3	Q96B67	TargetRank	High
DOWN	ARRDC3	Q96B67	TargetScan	Very High
DOWN	ARRDC3	Q96B67	miRDB	High
DOWN	ARRDC3	Q96B67	miRTar2GO	High
DOWN	ARRDC3	Q96B67	MirAncesTar	High
DOWN	ARRDC3	Q96B67	microrna.org	Very High
DOWN	BMT2	Q1RMZ1	Cupid	Very High
DOWN	BMT2	Q1RMZ1	DIANA	High

DOWN	**BMT2**	Q1RMZ1	ElMMo3	Very High
DOWN	**BMT2**	Q1RMZ1	Mirza-G	High
DOWN	**BMT2**	Q1RMZ1	PACCMIT	High
DOWN	**BMT2**	Q1RMZ1	TargetRank	High
DOWN	**BMT2**	Q1RMZ1	TargetScan	Very High
DOWN	**BMT2**	Q1RMZ1	miRDB	High
DOWN	**BMT2**	Q1RMZ1	miRTar2GO	High
DOWN	**BMT2**	Q1RMZ1	microrna.org	Very High
DOWN	**BSG**	P35613	microrna.org	High
DOWN	**BTG1**	P62324	microrna.org	Very High
DOWN	**BTG2**	P78543	CoMeTa	High
DOWN	**BTG2**	P78543	Cupid	Very High
DOWN	**BTG2**	P78543	DIANA	High
DOWN	**BTG2**	P78543	ElMMo3	Very High
DOWN	**BTG2**	P78543	MultiMiTar	High
DOWN	**BTG2**	P78543	PACCMIT	High
DOWN	**BTG2**	P78543	TargetScan	Very High
DOWN	**BTG2**	P78543	miRTar2GO	High
DOWN	**CAV2**	P51636	CoMeTa	Very High
DOWN	**CAV2**	P51636	Cupid	Very High
DOWN	**CAV2**	P51636	DIANA	High
DOWN	**CAV2**	P51636	ElMMo3	Very High
DOWN	**CAV2**	P51636	MAMI	Very High
DOWN	**CAV2**	P51636	Mirza-G	High
DOWN	**CAV2**	P51636	PACCMIT	High
DOWN	**CAV2**	P51636	TargetScan	Very High
DOWN	**CAV2**	P51636	miRDB	High
DOWN	**CAV2**	P51636	miRTar2GO	High
DOWN	**CAV2**	P51636	microrna.org	Very High
DOWN	**CD109**	Q6YHK3	miRTar2GO	High
DOWN	**CDKN1A**	P38936	miRTar2GO	High
DOWN	**CDKN2D**	P55273	miRTar2GO	High
DOWN	**CELF2**	O95319	CoMeTa	Very High
DOWN	**CELF2**	O95319	Cupid	Very High
DOWN	**CELF2**	O95319	ElMMo3	Very High
DOWN	**CELF2**	O95319	Mirza-G	High
DOWN	**CELF2**	O95319	MultiMiTar	High
DOWN	**CELF2**	O95319	PACCMIT	High
DOWN	**CELF2**	O95319	TargetRank	Very High
DOWN	**CELF2**	O95319	TargetScan	Very High
DOWN	**CELF2**	O95319	miRTar2GO	High
DOWN	**CELF2**	O95319	microrna.org	Very High
DOWN	**CIRBP**	Q14011	microrna.org	High
DOWN	**CNR1**	P21554	CoMeTa	High
DOWN	**CNR1**	P21554	Cupid	Very High
DOWN	**CNR1**	P21554	ElMMo3	Very High

DOWN	CNR1	P21554	MultiMiTar	High
DOWN	CNR1	P21554	PACCMIT	High
DOWN	CNR1	P21554	TargetRank	Very High
DOWN	CNR1	P21554	TargetScan	Very High
DOWN	CNR1	P21554	MirAncesTar	High
DOWN	CNR1	P21554	microrna.org	High
DOWN	CYR61	O00622	miRTar2GO	High
DOWN	DMXL1	Q9Y485	MultiMiTar	High
DOWN	DMXL1	Q9Y485	microrna.org	High
DOWN	DNAJC5	Q9H3Z4	miRTar2GO	High
DOWN	DUSP4	Q13115	miRTar2GO	High
DOWN	EPHA7	Q15375	microrna.org	High
DOWN	EPS8L2	Q9H6S3	MAMI	High
DOWN	FAM117B	Q6P1L5	miRTar2GO	High
DOWN	FAM117B	Q6P1L5	microrna.org	High
DOWN	FAM46B	Q96A09	MAMI	High
DOWN	FGF7	P21781	microrna.org	Very High
DOWN	FRAT2	O75474	CoMeTa	High
DOWN	FRAT2	O75474	Cupid	Very High
DOWN	FRAT2	O75474	ElMMo3	Very High
DOWN	FRAT2	O75474	PACCMIT	High
DOWN	FRAT2	O75474	TargetRank	Very High
DOWN	FRAT2	O75474	TargetScan	Very High
DOWN	FRAT2	O75474	miRTar2GO	High
DOWN	FRAT2	O75474	MirAncesTar	High
DOWN	FRAT2	O75474	microrna.org	Very High
DOWN	GNG2	P59768	Cupid	Very High
DOWN	GNG2	P59768	ElMMo3	High
DOWN	GNG2	P59768	PACCMIT	High
DOWN	GNG2	P59768	TargetScan	Very High
DOWN	GNG2	P59768	microrna.org	High
DOWN	GPI	P06744	MirTar	High
DOWN	GPI	P06744	miRTar2GO	High
DOWN	GPI	P06744	microrna.org	High
DOWN	GRIA4	P48058	MultiMiTar	High
DOWN	GRIA4	P48058	microrna.org	High
DOWN	GYS1	P13807	microrna.org	High
DOWN	HBEGF	Q99075	CoMeTa	High
DOWN	HBEGF	Q99075	Cupid	Very High
DOWN	HBEGF	Q99075	DIANA	High
DOWN	HBEGF	Q99075	ElMMo3	Very High
DOWN	HBEGF	Q99075	MultiMiTar	High
DOWN	HBEGF	Q99075	TargetScan	Very High
DOWN	HBEGF	Q99075	miRTar2GO	High
DOWN	HBEGF	Q99075	microrna.org	High
DOWN	HBP1	O60381	CoMeTa	Very High

DOWN	**HBP1**	O60381	Cupid	Very High
DOWN	**HBP1**	O60381	DIANA	Very High
DOWN	**HBP1**	O60381	ElMMo3	Very High
DOWN	**HBP1**	O60381	MAMI	Very High
DOWN	**HBP1**	O60381	MirTar	High
DOWN	**HBP1**	O60381	Mirza-G	High
DOWN	**HBP1**	O60381	MultiMiTar	High
DOWN	**HBP1**	O60381	PACCMIT	High
DOWN	**HBP1**	O60381	TargetRank	Very High
DOWN	**HBP1**	O60381	TargetScan	Very High
DOWN	**HBP1**	O60381	miRDB	High
DOWN	**HBP1**	O60381	microrna.org	Very High
DOWN	**HERC3**	Q15034	microrna.org	High
DOWN	**HIGD1A**	Q9Y241	miRTar2GO	High
DOWN	**HLF**	Q16534	CoMeTa	High
DOWN	**HLF**	Q16534	ElMMo3	Very High
DOWN	**HLF**	Q16534	TargetRank	Very High
DOWN	**HLF**	Q16534	TargetScan	Very High
DOWN	**HLF**	Q16534	microrna.org	High
DOWN	**HOGA1**	Q86XE5	microrna.org	High
DOWN	**HOMER1**	Q86YM7	TargetScan	Very High
DOWN	**ID3**	Q02535	MultiMiTar	High
DOWN	**ID3**	Q02535	miRTar2GO	High
DOWN	**ID3**	Q02535	microrna.org	High
DOWN	**IER2**	Q9BTL4	miRTar2GO	High
DOWN	**ISLR2**	Q6UXK2	Cupid	Very High
DOWN	**ISLR2**	Q6UXK2	DIANA	High
DOWN	**ISLR2**	Q6UXK2	ElMMo3	Very High
DOWN	**ISLR2**	Q6UXK2	PACCMIT	High
DOWN	**ISLR2**	Q6UXK2	TargetScan	Very High
DOWN	**ISLR2**	Q6UXK2	MirAncesTar	High
DOWN	**ISLR2**	Q6UXK2	microrna.org	High
DOWN	**JUN**	P05412	microrna.org	High
DOWN	**KDM4B**	O94953	CoMeTa	High
DOWN	**KDM4B**	O94953	Cupid	Very High
DOWN	**KDM4B**	O94953	ElMMo3	High
DOWN	**KDM4B**	O94953	TargetScan	Very High
DOWN	**KLF11**	O14901	CoMeTa	High
DOWN	**KLF11**	O14901	miRTar2GO	High
DOWN	**KLF11**	O14901	microrna.org	Very High
DOWN	**KLF2**	Q9Y5W3	microrna.org	High
DOWN	**KLHL24**	Q6TFL4	ElMMo3	High
DOWN	**KLHL24**	Q6TFL4	MultiMiTar	High
DOWN	**LOX**	P28300	Cupid	Very High
DOWN	**LOX**	P28300	DIANA	High
DOWN	**LOX**	P28300	ElMMo3	Very High

DOWN	**LOX**	P28300	MirTar	High
DOWN	**LOX**	P28300	Mirza-G	High
DOWN	**LOX**	P28300	TargetScan	Very High
DOWN	**LOX**	P28300	miRDB	High
DOWN	**LOX**	P28300	miRTar2GO	High
DOWN	**LOX**	P28300	MirAncesTar	High
DOWN	**LOX**	P28300	microrna.org	High
DOWN	**MCTP2**	Q6DN12	microrna.org	High
DOWN	**MPP2**	Q14168	MultiMiTar	High
DOWN	**MXI1**	P50539	CoMeTa	Very High
DOWN	**MXI1**	P50539	Cupid	Very High
DOWN	**MXI1**	P50539	ElMMo3	Very High
DOWN	**MXI1**	P50539	TargetScan	Very High
DOWN	**MYO1H**	Q8N1T3	microrna.org	High
DOWN	**NAMPT**	P43490	microrna.org	Very High
DOWN	**NSUN3**	Q9H649	CoMeTa	High
DOWN	**OTUD1**	Q5VV17	Cupid	Very High
DOWN	**OTUD1**	Q5VV17	ElMMo3	High
DOWN	**OTUD1**	Q5VV17	microrna.org	High
DOWN	**PCGF5**	Q86SE9	MultiMiTar	High
DOWN	**PCGF5**	Q86SE9	miRTar2GO	High
DOWN	**PDK1**	Q15118	miRTar2GO	High
DOWN	**PDK1**	Q15118	microrna.org	High
DOWN	**PER1**	O15534	CoMeTa	High
DOWN	**PER1**	O15534	Cupid	Very High
DOWN	**PER1**	O15534	ElMMo3	Very High
DOWN	**PER1**	O15534	MAMI	High
DOWN	**PER1**	O15534	MultiMiTar	High
DOWN	**PER1**	O15534	PACCMIT	High
DOWN	**PER1**	O15534	TargetScan	Very High
DOWN	**PFKL**	P17858	miRTar2GO	High
DOWN	**PFKP**	Q01813	microrna.org	High
DOWN	**PI15**	O43692	CoMeTa	High
DOWN	**PI15**	O43692	Cupid	Very High
DOWN	**PI15**	O43692	DIANA	High
DOWN	**PI15**	O43692	ElMMo3	Very High
DOWN	**PI15**	O43692	MirTar	High
DOWN	**PI15**	O43692	Mirza-G	High
DOWN	**PI15**	O43692	MultiMiTar	High
DOWN	**PI15**	O43692	PACCMIT	Very High
DOWN	**PI15**	O43692	TargetRank	Very High
DOWN	**PI15**	O43692	TargetScan	Very High
DOWN	**PI15**	O43692	miRTar2GO	High
DOWN	**PI15**	O43692	MirAncesTar	High
DOWN	**PI15**	O43692	microrna.org	High
DOWN	**PIK3CB**	P42338	TargetScan	Very High

DOWN	**PNRC1**	Q12796	microrna.org	Very High
DOWN	**PRDM1**	O75626	CoMeTa	High
DOWN	**PRDM1**	O75626	Cupid	Very High
DOWN	**PRDM1**	O75626	ElMMo3	High
DOWN	**PRDM1**	O75626	miRTar2GO	High
DOWN	**PRDM1**	O75626	MirAncesTar	High
DOWN	**PRDM1**	O75626	microrna.org	High
DOWN	**PRKAB2**	O43741	CoMeTa	High
DOWN	**PRKAB2**	O43741	Cupid	Very High
DOWN	**PRKAB2**	O43741	DIANA	High
DOWN	**PRKAB2**	O43741	ElMMo3	Very High
DOWN	**PRKAB2**	O43741	MirTar	High
DOWN	**PRKAB2**	O43741	MultiMiTar	High
DOWN	**PRKAB2**	O43741	TargetRank	High
DOWN	**PRKAB2**	O43741	TargetScan	Very High
DOWN	**PRKAB2**	O43741	microrna.org	High
DOWN	**PTTG1IP**	P53801	miRTar2GO	High
DOWN	**PXMP4**	Q9Y6I8	CoMeTa	High
DOWN	**PXMP4**	Q9Y6I8	ElMMo3	High
DOWN	**PXMP4**	Q9Y6I8	MultiMiTar	High
DOWN	**PXMP4**	Q9Y6I8	TargetScan	Very High
DOWN	**PXMP4**	Q9Y6I8	MirAncesTar	High
DOWN	**RAP2C**	Q9Y3L5	microrna.org	Very High
DOWN	**RHOB**	P62745	CoMeTa	Very High
DOWN	**RNF217**	Q8TC41	TargetScan	Very High
DOWN	**RPS10**	P46783	MAMI	High
DOWN	**RPS24**	P62847	CoMeTa	High
DOWN	**RPS24**	P62847	miRTar2GO	High
DOWN	**RUSC2**	Q8N2Y8	miRTar2GO	High
DOWN	**SGK1**	O00141	CoMeTa	High
DOWN	**SGK1**	O00141	Cupid	Very High
DOWN	**SGK1**	O00141	ElMMo3	Very High
DOWN	**SGK1**	O00141	MAMI	High
DOWN	**SGK1**	O00141	MirTar	High
DOWN	**SGK1**	O00141	Mirza-G	High
DOWN	**SGK1**	O00141	MultiMiTar	High
DOWN	**SGK1**	O00141	PACCMIT	High
DOWN	**SGK1**	O00141	TargetScan	Very High
DOWN	**SGK1**	O00141	miRDB	High
DOWN	**SGK1**	O00141	miRTar2GO	High
DOWN	**SGK1**	O00141	MirAncesTar	High
DOWN	**SGK1**	O00141	microrna.org	Very High
DOWN	**SLC2A1**	P11166	CoMeTa	Very High
DOWN	**SRGAP3**	O43295	CoMeTa	High
DOWN	**SRGAP3**	O43295	MultiMiTar	High
DOWN	**SRGAP3**	O43295	MirAncesTar	High

DOWN	**TIPARP**	Q7Z3E1	CoMeTa	Very High
DOWN	**TNFRSF9**	Q07011	CoMeTa	High
DOWN	**TNFRSF9**	Q07011	Cupid	Very High
DOWN	**TNFRSF9**	Q07011	DIANA	High
DOWN	**TNFRSF9**	Q07011	MAMI	High
DOWN	**TNFRSF9**	Q07011	TargetRank	Very High
DOWN	**TNFRSF9**	Q07011	microrna.org	Very High
DOWN	**TPT1**	P13693	microrna.org	Very High
DOWN	**TXNIP**	Q9H3M7	miRTar2GO	High
DOWN	**UPK1B**	O75841	CoMeTa	High
DOWN	**UPK1B**	O75841	ElMMo3	High
DOWN	**UPK1B**	O75841	MAMI	High
DOWN	**UPK1B**	O75841	MultiMiTar	High
DOWN	**UPK1B**	O75841	PACCMIT	High
DOWN	**UPK1B**	O75841	TargetScan	Very High
DOWN	**UPK1B**	O75841	microrna.org	High
DOWN	**VIM**	P08670	CoMeTa	Very High
DOWN	**WISP2**	O76076	miRTar2GO	High
DOWN	**WWC1**	Q8IX03	MAMI	High
DOWN	**XDH**	P47989	ElMMo3	High
DOWN	**XDH**	P47989	TargetRank	Very High
DOWN	**XDH**	P47989	microrna.org	High
DOWN	**ZC3H6**	P61129	MultiMiTar	High
DOWN	**ZFP36**	P26651	CoMeTa	High
DOWN	**ZFP36**	P26651	Cupid	Very High
DOWN	**ZFP36**	P26651	ElMMo3	Very High
DOWN	**ZFP36**	P26651	MAMI	High
DOWN	**ZFP36**	P26651	TargetScan	Very High
DOWN	**ZFP36**	P26651	microrna.org	High

References:

[1] Rosano S, Cora D, Parab S, Zaffuto S, Isella C, Porporato R, Hoza RM, Calogero RA, Riganti C, Bussolino F, et al. (2020). A regulatory microRNA network controls endothelial cell phenotypic switch during sprouting angiogenesis *Elife* **9**.

[2] Tokar T, Pastrello C, Rossos AEM, Abovsky M, Hauschild AC, Tsay M, Lu R, Jurisica I (2018). mirDIP 4.1-integrative database of human microRNA target predictions *Nucleic Acids Res* **46**, D360-D370.
